# Structural and dynamic similarities of nanofibrils and microparticles of engineered spider silk proteins probed by solid‐state NMR spectroscopy

**DOI:** 10.1002/pro.70460

**Published:** 2026-01-20

**Authors:** Nina Wehr, Ettore Bartalucci, Sabrina Smid, Georg Künze, Martin Humenik, Thomas Scheibel, Thomas Wiegand

**Affiliations:** ^1^ Institute of Technical and Macromolecular Chemistry RWTH Aachen University Aachen Germany; ^2^ Magnetic Resonance of Complex Materials and Catalysts Max Planck Institute for Chemical Energy Conversion Mülheim Germany; ^3^ Institute for Drug Discovery Leipzig University Leipzig Germany; ^4^ Interdisciplinary Center for Bioinformatics Leipzig University Leipzig Germany; ^5^ Center for Scalable Data Analytics and Artificial Intelligence Leipzig University Leipzig Germany; ^6^ Department of Biomaterials University Bayreuth Bayreuth Germany; ^7^ Bayreuth Center of Material Science and Engineering University Bayreuth Bayreuth Germany; ^8^ Bavarian Polymer Institute University Bayreuth Bayreuth Germany; ^9^ Bayreuth Center of Colloids and Interfaces University Bayreuth Bayreuth Germany; ^10^ Bayreuth Center for Molecular Biosciences University Bayreuth Bayreuth Germany; ^11^ North Bavarian NMR‐Center University Bayreuth Bayreuth Germany

**Keywords:** polarization transfers, poly‐alanine, recombinant spider silk, secondary structures, self‐assembly

## Abstract

Spider silks are proteinaceous fiber materials inspiring material design in various technical and biomedical fields due to their exceptional toughness, which exceeds that of most natural and artificial fibers. Solid‐state nuclear magnetic resonance (NMR) spectroscopy has been used herein to obtain insights into the structure and dynamics of ^13^C/^15^N isotope‐labeled nanofibrils and microparticles made of the recombinantly produced, engineered spider silk protein eADF4(C16), for which structural information was still lacking. Although these two β‐sheet‐rich morphologies differ substantially in their microscopic appearance (nanofibrils vs. microparticles), the solid‐state NMR spectra reveal high structural and dynamic similarities at the atomic level. For both morphologies, it was found that the rigid alanine stretch in the eADF4 sequence forms a mixture of rectangular and staggered β‐sheets extending to the flanking serine residues. In addition, our data reveal that the tyrosine sidechains are rigidified, which suggests their engagement in π–π‐stacking interactions. All of the glutamic acid residues were found to be deprotonated, which implies their localization on the outside of the fibril, where their negative charge can be compensated. *Trans*‐ as well as *cis*‐conformations were observed for proline residues, which suggests that they might further control the formation and extension of the poly‐alanine β‐sheet region during the self‐assembly process. The gained understanding of structure, dynamics, and assembly of the engineered spider silk protein eADF4(C16) will enable the tailored design of functional spider silk‐based biomaterials in the future. It will be especially useful in context of chemical modifications and genetic fusions supporting the development of fibril‐based hydrogel systems in the field of biosensing and tissue engineering.

## 1 INTRODUCTION

Spider silk fibers inspire material scientists due to their outstanding toughness resulting from the combination of elasticity and tensile strength, outperforming a vast majority of natural and artificial materials (Gosline et al., 1984; Lewis, 2006). Moreover, the lack of toxicity, low immunogenicity, as well as biodegradability underline the biocompatibility of spider silks. However, due to the very territorial and cannibalistic behavior of spiders, farming them on an industrial level similar to silkworms is not possible (Fox, 1975). These obstacles in spider silk production on large scales motivated the development of recombinant spider silk technologies allowing scalable production of engineered spider silk protein variants in various host organisms (Breslauer, 2024; Scheibel, 2004). In particular, recombinant spider silk is of high importance for biomedical applications, since it guarantees constant quality in contrast to that of natural silks (Wenk et al., 2011). Beyond processing of high performing fibers (Heidebrecht et al., 2015; Saric et al., 2021), recombinant spider silk proteins can be used to prepare diverse morphologies, such as nanofibrils (Humenik et al., 2015a; Slotta et al., 2007), nano‐ and microparticles (Elsner et al., 2015; Lammel et al., 2008), electrospun meshes (Leal‐Egaña et al., 2012; Sommer & Scheibel, 2023), foams (Schacht et al., 2016), coatings (Lentz et al., 2022), or 3D‐printable nanofibril‐based hydrogels (Ng et al., 2025; Schacht et al., 2015). Their potential in technical (Michel & Scheibel, 2025) as well as in biomedical applications including cardiac tissue engineering (Petzold et al., 2017), drug delivery systems (Herold et al., 2020; Kumari et al., 2018), biofabrication (Lechner et al., 2022; Steiner et al., 2021), microtissue engineering (Heinritz et al., 2022), or antimicrobial surface engineering (Kumari et al., 2020; Mohotti et al., 2025) has been reported.

**FIGURE 1:**
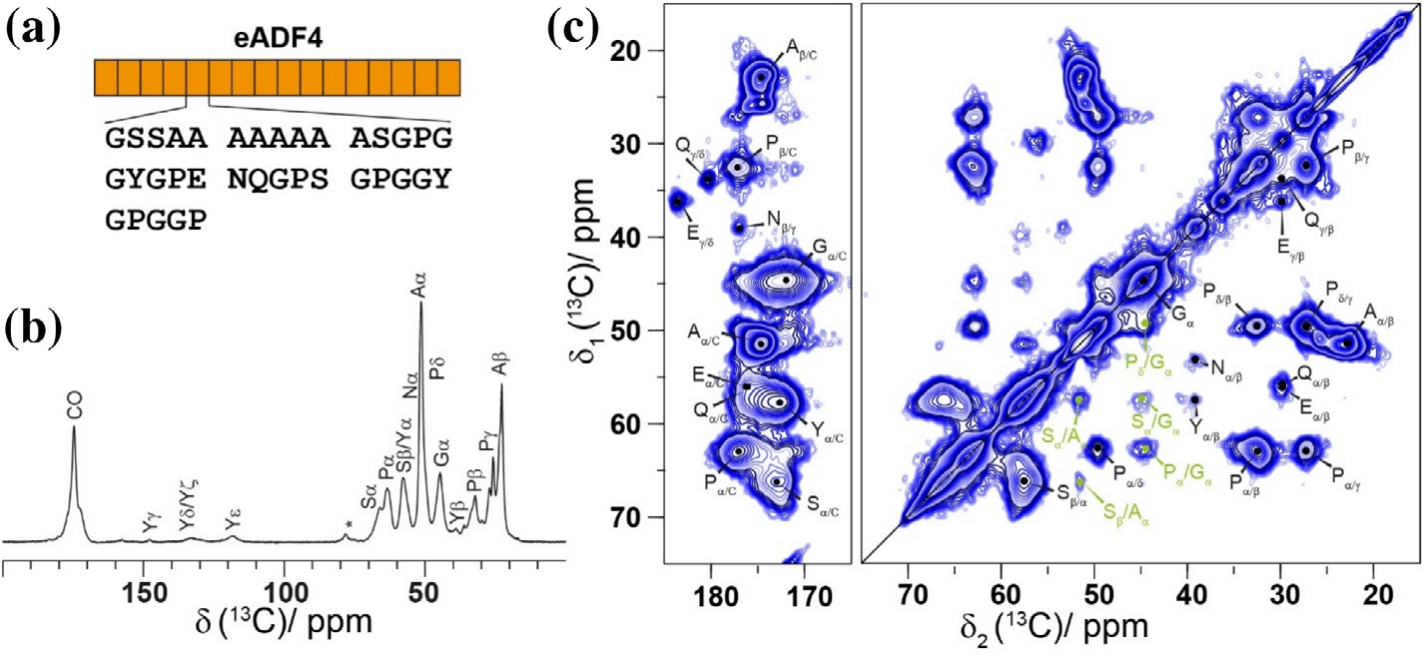
CP‐based spectra of fibrils identify all amino acid residues that are located in the rigid fibril core. (a) Schematic representation of the 16 repeat units of the eADF4(C16) construct and the amino acid sequence of the repeat motif. (b) 1D ^1^H‐^13^C CP‐MAS spectrum of the C16‐F sample with the corresponding resonance assignments. *MAS sideband. (c) ^13^C‐^13^C 2D 20 ms DARR spectrum of C16‐F with the corresponding resonance assignments. Inter‐residual cross peaks are marked in green. All spectra have been recorded at 16.4 T using a MAS frequency of 17.0 kHz.

In this study, we focus on the engineered spider silk protein eADF4(C16), which is based on the major ampullate silk fibroin 4 of *Araneus diadematus* (ADF4). The recombinant protein consists of 16 repeat units (see Figure 1a for the amino acid sequence) (Huemmerich et al., 2004). Depending on the concentration of the kosmotropic phosphate (Pi) ions used, the protein self‐assembles into nanofibrils (<400 mM Pi) or forms regularly shaped spherical particles via a salting‐out process (>400 mM Pi) (Lammel et al. 2008; Slotta et al. 2007). The formation of fibrils is a nucleation‐dependent process (Humenik et al., 2015a), including primary and secondary nucleation (Hovanová, Hovan, Žoldák, et al., 2023). Interestingly, the assembly rates decrease for the truncated protein variants eADF4(Cn) (with *n* = 8, 4, 2, 1), which highlights the importance of intermolecular protein folding into a β‐sheet‐rich structure triggering the protein oligomerization and nucleus formation (Humenik et al., 2014). The final fibrils show a typical cross‐β X‐ray diffraction pattern and bind molecular probes such as thioflavin T or Congo red similar to amyloid fibrils (Humenik et al., 2014; Slotta et al., 2007). The nanofibrils have been used as building blocks for nanohydrogel coatings (Humenik et al., 2020; Zha et al., 2019) as well as for physically cross‐linked printable hydrogels (Lechner et al., 2022; Schacht et al., 2015). Particles made of eADF4(C16) comprise high content of β‐sheets, similarly to the fibrils, and can be described as round‐shaped structures. The sizes of the particles can be adjusted between 200 nm and 10 μm (Lammel et al., 2008). These microparticles are especially interesting as potential drug delivery systems (Helfricht et al., 2013; Humenik et al., 2011; Schierling et al., 2016), fillers for spider silk hydrogels modifying the drug release kinetics (Kumari et al., 2018) or collagen hydrogels for printability and improved stability (Ng et al., 2025). Interestingly, the eADF4(C16) particles show polymer‐brush‐like surface properties (Helfricht et al., 2013), which allow nucleation of nanofibril growth of the same protein (Humenik et al., 2015a).

Solid‐state nuclear magnetic resonance (NMR) is a suitable method for the investigation of solid, amorphous, and strongly disordered materials due to the high sensitivity of NMR observables to the local environment, even in compounds entirely lacking long‐range order (Ku et al., 1996; Siemer, 2020). It has previously been intensively used to investigate the structure of major ampullate spider silk (Asakura & Naito, 2024) showing that the polyalanine (poly‐Ala) region, as a central element in the repetitive motifs, forms β‐sheet crystallites (Guerette et al., 1996). For proteins, the sensitivity of ^13^C NMR chemical‐shift values on the dihedral angles can be used to access information on the secondary structure (Wang & Jardetzky, 2002). Furthermore, the NMR observables are affected by noncovalent interactions, such as hydrogen bonds (for a recent perspective article see [Schröder et al., 2024]). Those play an important role in β‐sheet formation in the crystalline region of natural spider silk, which determines their toughness (Papadopoulos et al., 2009; Vollrath & Porter, 2006). For eADF4(C16), ^13^C‐detected magic angle spinning (MAS) spectra of films have been previously recorded to identify the conformation of the protein in the film, and the study revealed that the poly‐Ala sequence forms an α‐helix conformation in non‐treated and a β‐sheet conformation in primary alcohol post‐treated films (Spiess et al., 2011). In case of self‐assembled fibrils and salted‐out particles, the presence of the abundant β‐sheets as well as less structured regions have been confirmed using CD‐ and FT‐IR spectroscopy (Humenik et al., 2015a; Slotta et al., 2008), however the secondary structure elements were not assigned to specific residues yet (Creager et al., 2010). In this work, solid‐state NMR spectroscopy was applied to elucidate structural and dynamic properties of eADF4(C16) in nanofibrillar (further called C16‐F) and particulate (further C16‐P) states, highlighting their atomic‐level structural similarities despite their entirely different morphologies.

## 2 RESULTS AND DISCUSSION

### 2.1 Basic structural characteristics of fibrils

Nanofibrils of eADF4(C16) were characterized by solid‐state NMR spectroscopy using a ^13^C/^15^N isotope‐labeled protein sample. If not stated otherwise, C16‐F samples have been self‐assembled with 150 mM KPi. To identify the rigid fraction of the nanofibrils, ^1^H‐^13^C cross polarization (CP) experiments were conducted under magic‐angle spinning (MAS) conditions (Figure 1b). The CP polarization transfer is efficient for rigid fragments of the protein sample possessing slow isotropic molecular motion, with correlation times τ_c_ larger than 10^−5^ s (Aebischer & Ernst, 2024). In the ^13^C CP spectra, the carbonyl resonances appear at around 180 ppm, the signals ranging between 110 to 150 ppm can be assigned to the aromatic rings of the Tyr residues, and the resonances between 10 to 70 ppm aliphatic carbon atoms. In general, the ^13^C resonances in the CP spectrum of C16‐F are rather broad with a full‐width‐at‐half‐maximum (FWHM) linewidth of approximately 925 Hz for nanofibrils and 728 Hz for particles using the Gly Cα resonance as an example. This is comparable to the linewidths of previously reported solid‐state NMR spectra of recombinant as well as natural spider silks, where the broad resonances have been attributed to conformational disorder of the silk protein (Creager et al., 2010; Holland et al., 2008; Tasei et al., 2017). In the case of C16‐F, the large ^13^C linewidths might be attributed to structural differences among the 16 repeat units, as well as additional structural heterogeneities (e.g., conformational disorder), since the resonances are mostly inhomogeneously broadened. Specifically, the ^13^C inhomogeneous linewidth (Δ^inhomo^) is determined to around 605 Hz (calculated by subtracting the homogeneous linewidth obtained from spin‐echo decay curves from the total linewidth) for nanofibrils and to around 440 Hz for particles (as calculated from the Gly Cα of the respective 1D ^13^C CP spectra).* Interestingly, slightly less inhomogeneous broadening caused by conformational disorder is observed in case of the particles. To investigate whether the broadening stems from structural differences among the 16 repeat units, a shorter ^13^C/^15^N isotope‐labeled construct with only two C‐units (Humenik et al., 2014) (further C2‐F) has been prepared. The linewidth in ^13^C MAS spectra of this construct however remained similar to C16‐F (Figure S1) allowing to conclude that conformational disorder is independent of the number of C‐motifs. The fibril‐formation process of these eADF4(n) fibrils has previously been described as a cooperative event leading to an increase in assembly rates with increasing number of repeat units used in the construct. The different fibril formation kinetics, however, resulted in morphologically and structurally similar fibrils (Humenik et al., 2014), which is confirmed with our NMR‐spectroscopic findings showing very similar conformational disorder in the C2‐F and C16‐F samples.

Additionally, a 2D ^13^C‐^13^C dipolar‐assisted rotational resonance (DARR) spectrum was recorded (Figure 1c) (Takegoshi et al., 2001). This experiment uses a ^1^H‐^13^C CP step as an initial polarization transfer and, therefore, only probes rigid protein segments. Using the short mixing time (20 ms), mostly directly bound ^13^C‐^13^C spin pairs are observed, while a longer mixing time (200 ms) can be applied to additionally observe inter‐residual contacts (Figure S2 for C16‐F self‐assembled in the presence of 50 mM KPi). Therefore, the type of amino acid residues can be identified from such a spectrum. In the DARR spectrum of C16‐F, all amino acid residues present in the sequence were found and are thus part of the rigid protein fraction, i.e., part of the folded structure. The most intense intramolecular cross‐peaks in the spectrum can be assigned to Ala and Ser residues (Cα/Cβ chemical‐shift values of 51.5/22.8 ppm and 57.5/66.2 ppm, respectively). These amino acids as well as Pro and Gly residues show additional inter‐residue cross peaks, in agreement with the *i* → *i* ± 1 correlations expected from the amino‐acid sequence.

### 2.2 Dynamic NMR spectral editing of fibrils

**FIGURE 2:**
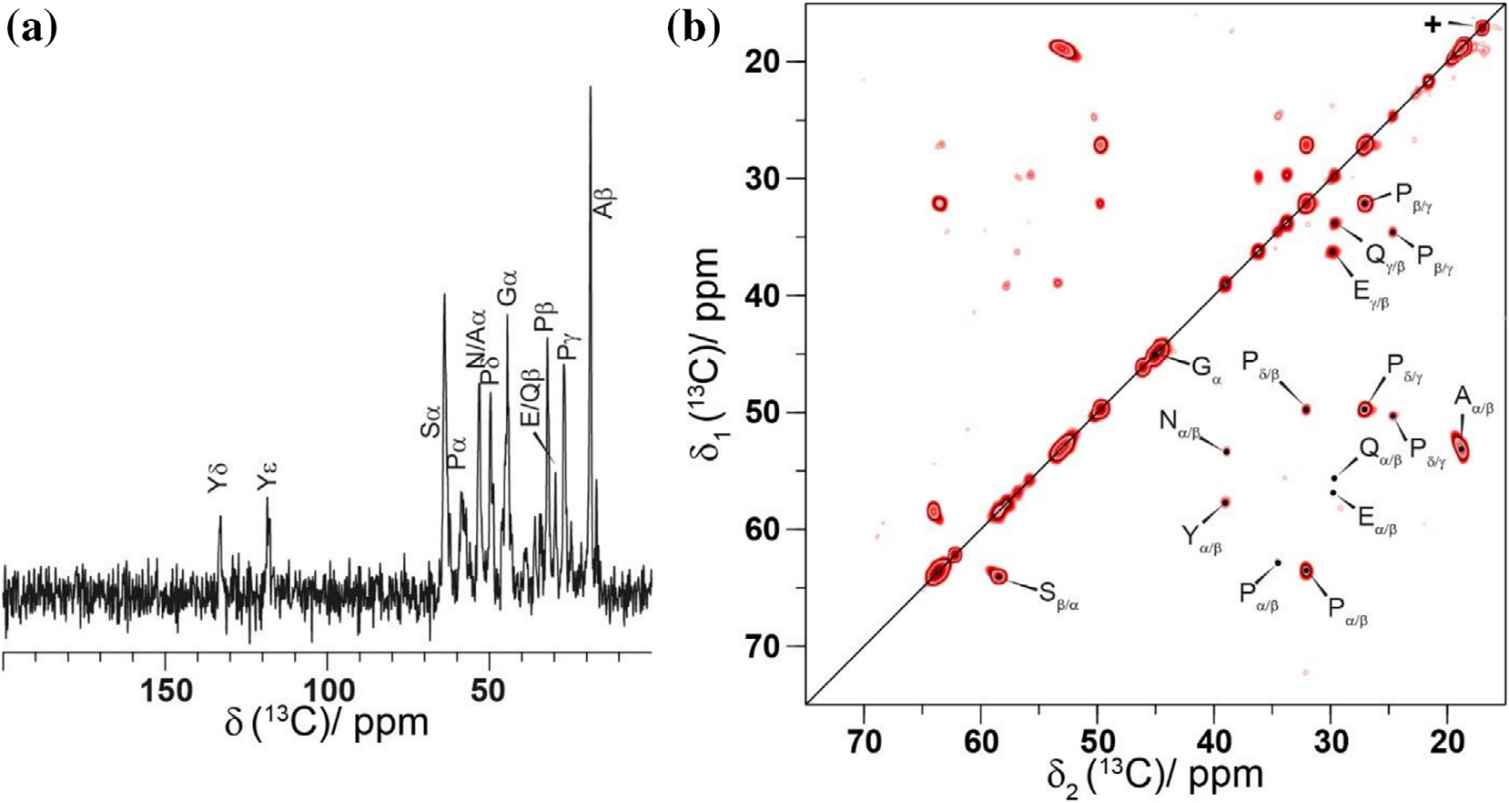
INEPT‐based experiments show all amino acid residues that are involved in highly mobile regions of the fibril. (a) 1D ^1^H‐^13^C INEPT spectrum of C16‐F including the resonance assignment. (b) 2D ^13^C‐^13^C INEPT‐TOBSY spectrum with a mixing time of 7.8 ms. For the TOBSY mixing the symmetry‐based ${P9}_{3}^{1}$ sequence was used. All spectra have been recorded at 16.4 T using an MAS frequency of 17.0 kHz. The resonance marked with a + on the diagonal is assigned to Met residues, which are part of the used tag comprising MASMT GGQMG RGSM.

Solid‐state NMR experiments can be used to distinguish between rigid and flexible segments of a protein in case of isotropic molecular motion. As mentioned above, the rigid parts can be detected by using CP‐based experiments. Insensitive Nuclei Enhanced Polarization Transfer (INEPT) experiments, in contrast, can be used to identify highly mobile parts of the sample experiencing fast isotropic motion with correlation times of less than 10^−7^ s (Aebischer & Ernst, 2024; Topgaard, 2023). This dynamic spectral editing has already been applied in several examples in the context of spider silk proteins (Asakura & Naito, 2024; Asakura, Tasei, Aoki, & Nishimura, 2018; Tasei et al., 2017; Wu et al., 2024). For C16‐F, ^1^H‐^13^C refocused INEPT signal is observed in addition to the CP signal discussed above (Figure 2a). This indicates that the C16‐F sample contains not only a highly rigid protein segment (probably a folded segment in the fibril core) as observed by CP, but also mobile segments. To obtain a resonance assignment of the observed INEPT signals, a 2D ^13^C‐^13^C INEPT total through‐bond correlation spectroscopy (TOBSY) experiment was recorded (Figure 2b, Figure S3) (Andronesi et al., 2005). The magnetization transfer in the INEPT‐TOBSY experiment is based on scalar *J*‐couplings. The cross peaks in such a spectrum, therefore, arise from directly‐bound carbons mediated via the homonuclear ^13^C‐^13^C *J*‐coupling. In the INEPT‐TOBSY spectrum, all amino acid residues present in the sequence are detected as well, indicating a highly mobile protein portion. Interestingly, three distinct glycine resonances at 44.4, 45.1, and 46.0 ppm could be identified, which might be explained by the different amino acids flanking such Gly residues, since the Gly Cα random coil chemical‐shift value is highly sensitive to such flanking residues. For example, in the case of a Pro preceding a Gly residue, a significant shielding of the Cα resonance is observed (Schwarzinger et al., 2001). Therefore, we assign the ^13^C Gly Cα resonance at 44.4 ppm to the proline‐glycine (PG) motifs. These PG motifs occur four times in the repeat unit and correspond to Gly1, Gly15, Gly28, and Gly33. The further two Gly resonances could not be assigned to a specific motif based on the chemical‐shift value. The observation of all amino‐acid types in the INEPT spectra could result from highly flexible protein parts within the fibrils or free monomers in the supernatant. The latter is, however, rather unlikely as supported by ^1^H and ^13^C solution‐state NMR spectra recorded on the supernatant remaining after sedimentation, which showed no signal. The assembly continues until complete depletion of monomers, consistent with previous observations obtained by ultracentrifugation (Humenik et al., 2014; Humenik et al., 2015a).

### 2.3 Assignment of amino acid residues to β‐sheet structures

**FIGURE 3:**
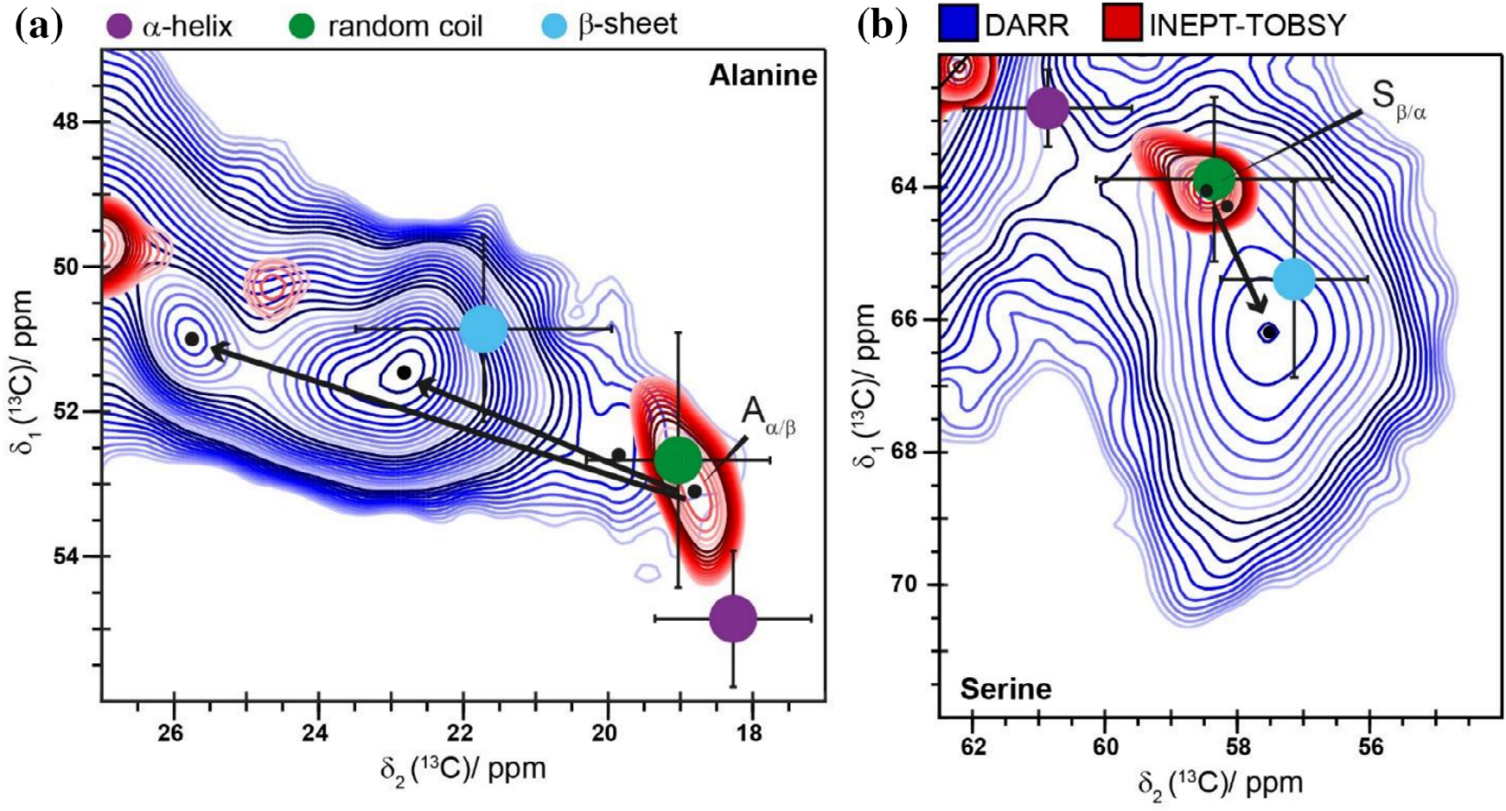
The chemical‐shift values of Ala and Ser Cα/Cβ cross peak differ significantly between the rigid and the mobile fraction of C16‐F, indicating the presence of different secondary structures between them. (a) Ala Cα/Cβ region and (b) Ser Cα/Cβ region in the ^13^C‐^13^C DARR spectrum (blue) and the ^13^C‐^13^C INEPT‐TOBSY spectrum (red). The typical regions of Cα/Cβ cross peaks according to Wang and Jardetzky for α‐helical, β‐sheet and random coil structures are plotted as dots in purple, light blue and green, respectively, with the corresponding standard deviations as error bars (Wang & Jardetzky, 2002). The black arrows indicate the difference in chemical shift between the mobile and the rigid fraction of C16‐F.

NMR spectroscopy is an ideal tool for identifying the secondary structure, since the ^13^C chemical‐shift values of the Cα and Cβ amino acid resonances are sensitive to the backbone dihedral angles Φ and Ψ (Ikura et al., 1991). To this end, the average chemical‐shift values and associated standard deviation for α‐helices, β‐sheets and random coils were taken from Wang and Jardetzky and compared to the experimentally observed ones (note that all our ^13^C chemical‐shift values are reported on the DSS scale) (Wang & Jardetzky, 2002). The Ala residues in the DARR experiment show three distinct Cα/Cβ cross peaks. The most intense correlation peaks at 51.5/22.8 ppm and at 51.0/25.9 ppm belong to Ala residues in two different β‐sheet structures. The third minor peak at 53.2/19.5 ppm, however, belongs to Ala in a random coil conformation (Figure 3a, green). The assignment of the downfield shifted peak at 51.0/25.1 ppm to an Ala Cα/Cβ correlation and not to a Pro Cδ/Cγ correlation was achieved by 2D NCa spectra for C16‐F self‐assembled with 50 mM KPi and C16‐P (Figure S4). In the INEPT‐TOBSY spectrum (Figure 3a, red), only the random coil fraction of Ala with a cross peak at 53.1/18.8 ppm is observed. Therefore, in the mobile section of the fibril, none of the Ala residues are forming a β‐sheet structure, as expected for an unfolded protein. Consequently, the secondary structure of Ala residues changes significantly during the fibril formation process. Hence, hydrogen bond formation during the formation of Ala β‐sheets is a central process contributing to the self‐assembly of C16‐F. As mentioned above for Ala in β‐sheet conformation, two different cross peaks were observed in the DARR spectrum. Those can be assigned to two different packing arrangements of the β‐sheets as previously reported for the spider dragline silk fiber from *Nephila clavata* as well as in short polypeptides (Asakura et al., 2012; Asakura, Tasei, Aoki, & Nishimura, 2018; Asakura, Tasei, Matsuda, & Naito, 2018). The cross peak at 51.5/22.8 ppm can thus be assigned to a rectangular β‐sheet, while the cross peak at 51.0/25.9 ppm arises from a staggered β‐sheet structure (Asakura et al., 2012). Moreover, both Ala cross peaks are heterogeneously broadened for the different β‐sheet structures, which indicates conformational disorder of the rectangular as well as the staggered β‐sheets, which are only poorly aligned. This was also observed for the dragline spider silk of *Trichonephila clavipes* (Simmons et al., 1996). Likewise, the Ser residues show a similar behavior. In the DARR spectrum (Figure 3b, blue) the major fraction of Ser residues occupies a β‐sheet conformation (Cα/Cβ cross peak at 57.5/66.2 ppm). Only a minor fraction of the rigid fibril adopts a random coil conformation (cross peak at 58.1/64.1 ppm). In the mobile fraction as probed by INEPT‐TOBSY (Figure 3b, red), only a random coil structure with a cross peak at 58.3/63.9 ppm is detected. We thus conclude that the β‐sheet structure of the fibril includes not only Ala residues, but also some of the flanking Ser residues.

### 2.4 The Glu sidechain is deprotonated

**FIGURE 4:**
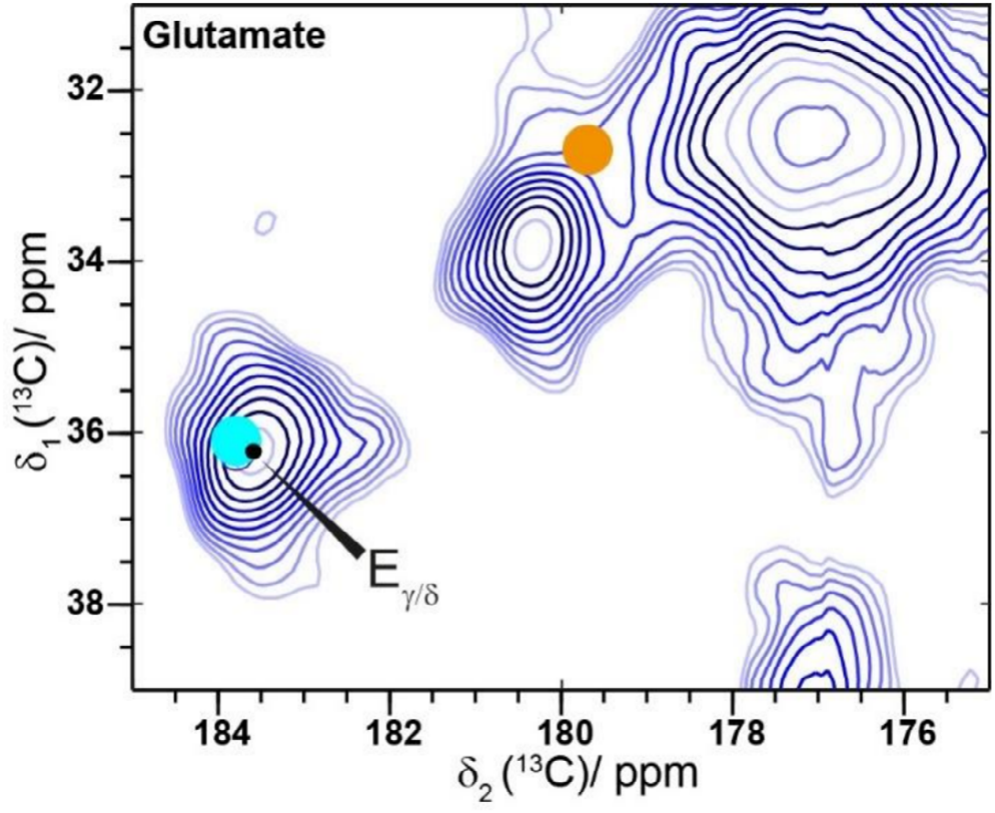
The chemical‐shift value of the glutamate Cδ resonance points to deprotonated glutamate residues in fibrils. Glutamate Cδ region of the ^13^C‐^13^C DARR 20 ms spectrum. The light blue circle marks the expected chemical shift of a deprotonated glutamate Cδ. The orange circle represents the expected chemical shift of a protonated glutamate Cδ (Platzer et al., 2014).

In addition to secondary structure information, the ^13^C chemical‐shift values can be used to determine the protonation state of Glu residues. A deprotonated Glu Cδ atom exhibits a more deshielded ^13^C resonance with an expected Cγ/Cδ cross peak at 36.1/183.8 ppm (cyan circle in Figure 4) than a protonated one with an expected cross peak at 32.7/179.7 ppm (orange circle in Figure 4). The observed chemical‐shift value for Cδ of 183.6 ppm in this case matches well with a deprotonated Glu (Platzer et al., 2014). Since the eADF4(C16) construct contains only two positively charged amino acid residues (amino terminus, and Arg‐residue in T7‐tag), which cannot compensate for the negative charge of 16 Glu residues, these sidechains in the fibril are most probably oriented outwards, that is, on the fibril surface. This is reflected in the colloidal stability of fibrils as well as the availability of Glu residues for chemical modifications, as demonstrated recently using *N*‐(3‐dimethylaminopropyl)‐*N*′‐ethylcarbodiimide (EDC)‐mediated covalent coupling of amino‐modified oligonucleotides (Lamberger et al., 2022).

### 2.5 Rigid Tyr resonances point to stabilizing π–π interactions

**FIGURE 5:**
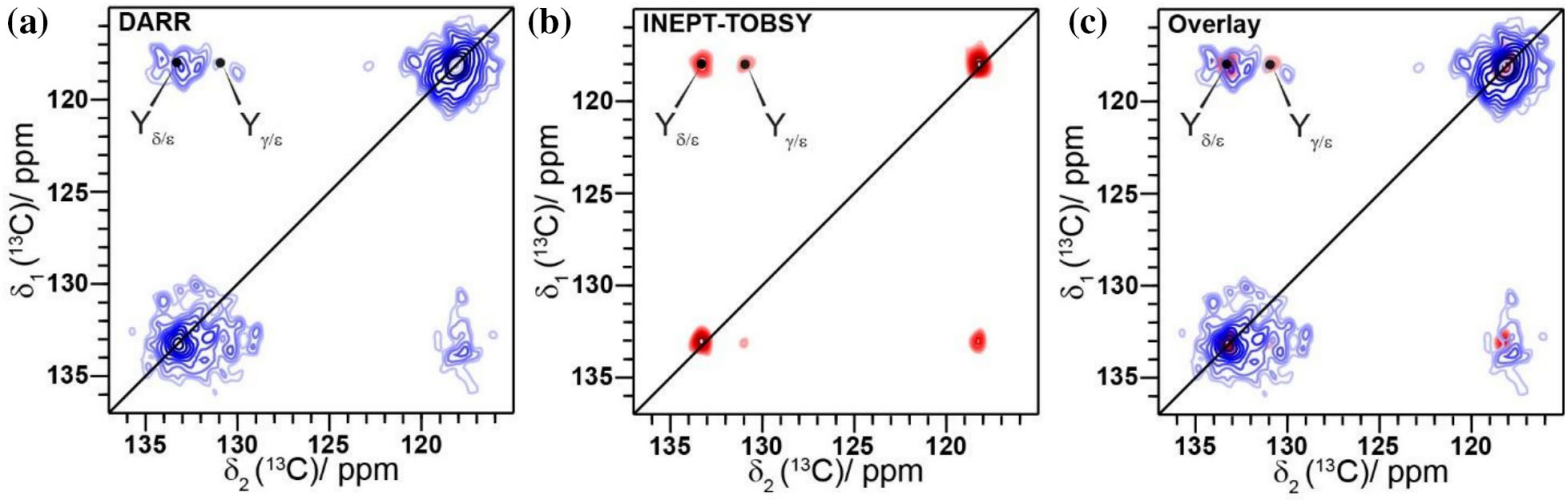
The aromatic ring of Tyr is part of the rigid fibrillar structure. (a) ^13^C‐^13^C DARR spectrum, (b) INEPT‐TOBSY spectrum and (c) an overlay of both spectra from (a) and (b) of the aromatic region, showing the Tyr sidechain resonances.

Another feature of the DARR spectrum is the appearance of the aromatic Tyr sidechain resonances at 118.1, 131.0, 133.4, and 157.7 ppm for C_ε_, C_γ_, C_δ_, and C_ζ_, respectively (Figure 5, Figure S5), consistent with a rigidified Tyr sidechain, for instance caused by π–π‐stacking interactions. These interactions are known to be important for the formation of amyloid fibrils (Gazit, 2002). In addition, CH–π interactions between Pro and Tyr residues have been observed in natural spider silk fibers (Chalek et al., 2024). The importance of Tyr residues in spider silk assembly has also been illustrated for recombinant spider silks, for which structural differences for the Tyr residues, such as different aromatic ring packings or hydrogen‐bonding differences, between dopes and fibers have been reported (Stengel et al., 2023). Tyr is also important during the spinning process of natural spider silks, where its ability to engage in π–π and π–OH interactions leads to a templating effect, which is enabling the formation of the crystalline parts of the spider silk (Partlow et al., 2016).

### 2.6 Pro residues adopt *cis*‐ and *trans*‐conformations

**FIGURE 6:**
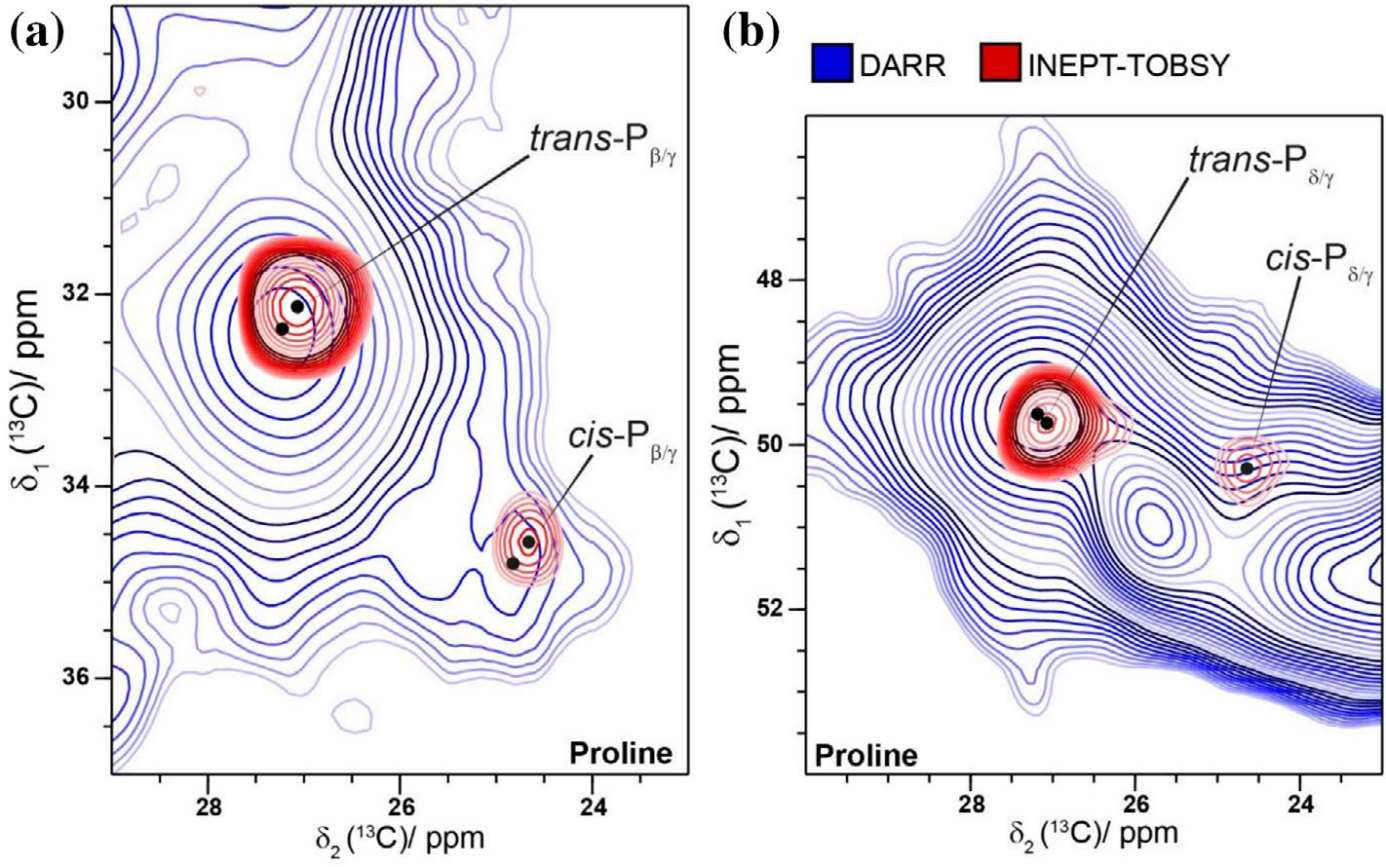
Two different sets of resonances of Pro belonging to *trans*‐ and *cis*‐configurations are observed. (a) Region with Cβ/Cγ cross peaks of Pro and (b) region with Cδ/Cγ of Pro in the ^13^C‐^13^C DARR spectrum (blue) and the ^13^C‐^13^C INEPT spectrum (red).

For the Pro residues, two different spin systems are observed in the DARR as well as in the INEPT‐TOBSY spectra (Figure 6a,b). The two Pro populations correspond to the typically more common *trans*‐Pro conformation and the less common *cis*‐Pro conformation. This is supported by the characteristic ^13^C chemical‐shift values, which agree with previous reported ones (Poznański et al., 1993). Integration of the 1D slices of the well‐separated Pγ resonances yielded an approximate *cis*:*trans* ratio of 1:3, which is higher than the Pro *cis*:*trans* ratio that was previously reported for the recombinant (AQ)_12_NR3 spider silk protein (Stengel et al., 2023), although the integration of the cross peaks in DARR spectra should be taken with some care as, for instance, the CP transfer is not quantitative.

The *cis/trans* isomerization is known to be important for the fibrillization of amyloid fibrils and has also been reported for β2 microtubullin (Torbeev & Hilvert, 2013), α‐synuclein (Meuvis et al., 2010), stefin B (Taler‐Verčič et al., 2017) as well as tau peptides (Jiji et al., 2016). For prions, however, Pro residues which are flanking β‐sheet structures are known to stop the extension of β‐sheets (Shamsir & Dalby, 2007). In eADF4(C16), all Pro residues are preceded by glycines, leading to the presence of a Gly‐*cis*Pro motif. For recombinant spider silk proteins, the Gly‐*cis*Pro motif is known to increase water exposed domains upon supercontraction. This Gly‐*cis*Pro motif is therefore important for the mechanical properties of spider silk proteins (Tasei et al., 2017). For other proteins, the Gly‐*cis*Pro motif has been shown to preserve β‐sheet formation from the distortions normally caused by *cis*Pro residues, wherefore they can often be found in β‐sheet structures (Das & Basu, 2012). These effects might play a role for eADF4(C16) as well, although the exact role of the *cis*Pro in eADF4(C16) remains elusive.

### 2.7 Structural modeling by using AlphaFold

**FIGURE 7:**
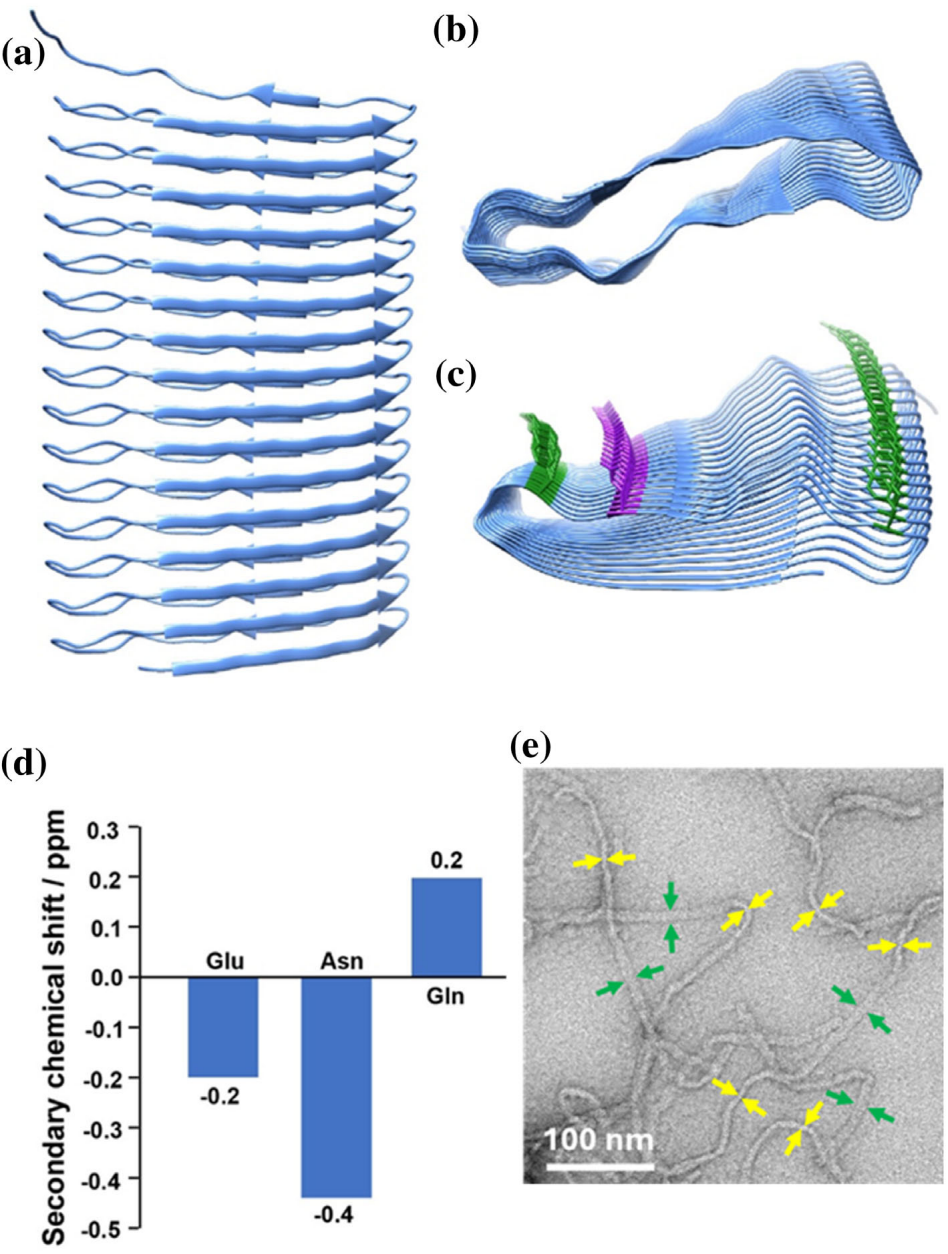
The AlphaFold3 model of eADF4(C16) shows a putative folded structure with the repeating poly‐Ala sequence forming a parallel β‐sheet. (a) The AlphaFold3 model of eADF4(C16), where the Ala stretches and the flanking Ser residues on both sites are forming a β‐sheet structure. In (b) and (c), the model is displayed from the top. In (c) Tyr side chains (green), which are engaged in π–π‐stacking interactions as well as glutamic acid side chains (purple) are highlighted, which face to the outside of the folded core. (d) ^13^C secondary chemical‐shift value plot for the residues Glu20‐Gln22, which appear only once per repeat and can therefore be assigned. (e) Visualization of the eADF4(C16) fibrils revealing ribbon like character in the 2D projection using TEM. Broad regions (green arrows) and narrowed regions (yellow arrows) are based on turns of the ribbon (adapted from reference Hovanová, Hovan, Humenik, and Sedlák [2023]).

We next used AlphaFold3 (Abramson et al., 2024) to generate a structural model of the eADF4(C16) protein (Figure 7). In addition, we created shorter models of eADF4 with the number of repeat units ranging from 2 to 15 (C2–C15) to investigate how the model confidence changes with varying repeat numbers (Figures S6 and S7). The confidence of the generated models is judged based on the predicted local distance difference test (pLDDT) and the predicted template modeling (pTM) score (Abramson et al., 2024). While these values are rather low for eADF4(C16) with pLDDT and pTM values of 56.4 and 43, significantly higher values and, thus, more reliable structural models are obtained for shorter protein constructs, except for eADF4(C2) and eADF4(C3) for which even lower values are obtained. Therefore, while the predicted backbone geometry of the different eADF4 models can be considered reliably converged, side‐chain orientations should be interpreted with caution in this confidence range, and conclusions based on specific side‐chain interactions must be viewed as qualitative rather than quantitative. The structures with the highest pLDDT and pTM values were obtained for eADF4(C5) and eADF4(C7), which have almost identical structures. A difference between eADF4(C5) and eADF4(C7) lies in the sidechain orientation of three Ser residues (Ser2, Ser3, and Ser25), which are directed either inside or outside of the fibril core (Figure S8). The Ser sidechain direction observed for the C5 structural model is the same as the one found for models with more repeats, such as eADF4(C16). Despite the lower score parameters, the AlphaFold3 model of eADF4(C16) is fairly similar to the C5 model (Figure S8) and can still be used as a qualitative structural model supporting several features observed in the experimental solid‐state NMR spectra (Figure 7). In particular, the Ala residues Ala4 to Ala11 are part of an elongated β‐sheet (determined by the STRIDE algorithm [Frishman & Argos, 1995]), which ranges from Ser3 to Ser12 in the AlphaFold model. In the eADF4 structure, the two β‐strands within each repeat fold back on one another, forming a compact β‐sandwich motif. Moreover, the β‐sheets of adjacent repeats align in an edge‐to‐edge manner, stabilized by backbone hydrogen bonding. Glu20 points to the outside of the fibrils as concluded from its protonation state (Figure 7c). The Tyr residues Tyr17 and Tyr30 indeed form Tyr ladders, in which the sidechains form intersheet π–π stacking, as concluded from their rigidification (see above). The second β‐sheet in the modeled structure (Glu20‐Gln22) is not confirmed by the secondary chemical‐shift values experimentally determined for these residues (see Figure 7d). Positive secondary chemical‐shift values indicate the formation of an α‐helix, while negative values indicate the formation of a β‐sheet, but accuracy is only achieved for three consecutive values showing the same trend with this method. Even though, based on the NMR data, some prolines were suggested to be in *cis*‐conformation, in the AlphaFold3 model all Pro residues are found in the more stable *trans*‐configuration. Although the model needs to be interpreted with caution, it supports many experimental findings and enables visualization of those in a plausible structural model.

The putative structure shown in Figure 7 can be interpreted in the context of previous observations on recombinant eADF4(Cn) variants, which do not comprise terminal domains and assemble into amyloid‐like nanofibrils. Assuming the proposed solenoid‐type β‐sheet architecture constitutes the fibril core through monomer stacking—either involving the full 16‐repeat fold (Figure 7a) or a partially folded ensemble (<16 repeats) consistent with the NMR indications of locally increased residue mobility—the dimensions of the predicted AlphaFold3 model align well with previous experimental data. AFM scans revealed fibril heights of approximately 2–2.5 nm, and TEM imaging has shown fibril diameters of 7–8 nm (Hovanová, Hovan, Žoldák, et al., 2023; Humenik et al., 2014; Humenik et al., 2015a; Humenik & Scheibel, 2014). The flat β‐solenoid architecture also supports the irregularly twisted ribbon‐like morphology observed previously in high‐resolution TEM imaging (Hovanová, Hovan, Humenik, & Sedlák, 2023) (Figure 7e). This solenoid architecture is also compatible with the presence of in‐register cross‐β structures, as previously inferred from x‐ray diffraction analyses of eADF4(C16) fibrils (Slotta et al., 2007), and which cannot be found in other major amputate spidroins. The high surface exposure of glutamic acid residues in the model (Figure 7c) indicates a strongly anionic fibril surface. Such electrostatic repulsion offers a plausible explanation for the absence of “maturation” into higher‐order fibrillar bundles herein as well as in the previous studies (Hovanová, Hovan, Žoldák, et al., 2023; Humenik et al., 2014; Humenik et al., 2015a; Humenik & Scheibel, 2014), a process characteristic for many amyloid‐like systems (Wei et al., 2017).

Importantly, the β‐solenoid fold is, however, quite specific for this explicit protein and highly atypical for other recombinant and native spider silk proteins, which more commonly feature antiparallel β‐sheet architectures and stacked β‐sheet nanocrystals thereof within their hierarchical fiber structure or other processed morphologies (Asakura, 2024; Du et al., 2006; Krishnaji et al., 2013; Numata & Kaplan, 2011; Oroudjev et al., 2002). In contrast, the current model highlights a distinct structural class within the broader landscape of silk‐assemblies, which resembles β‐solenoid motifs known from certain prion‐derived proteins and peptides (Flores‐Fernández et al., 2018).

### 2.8 Nanofibrils and microparticles reveal similar NMR spectra

**FIGURE 8:**
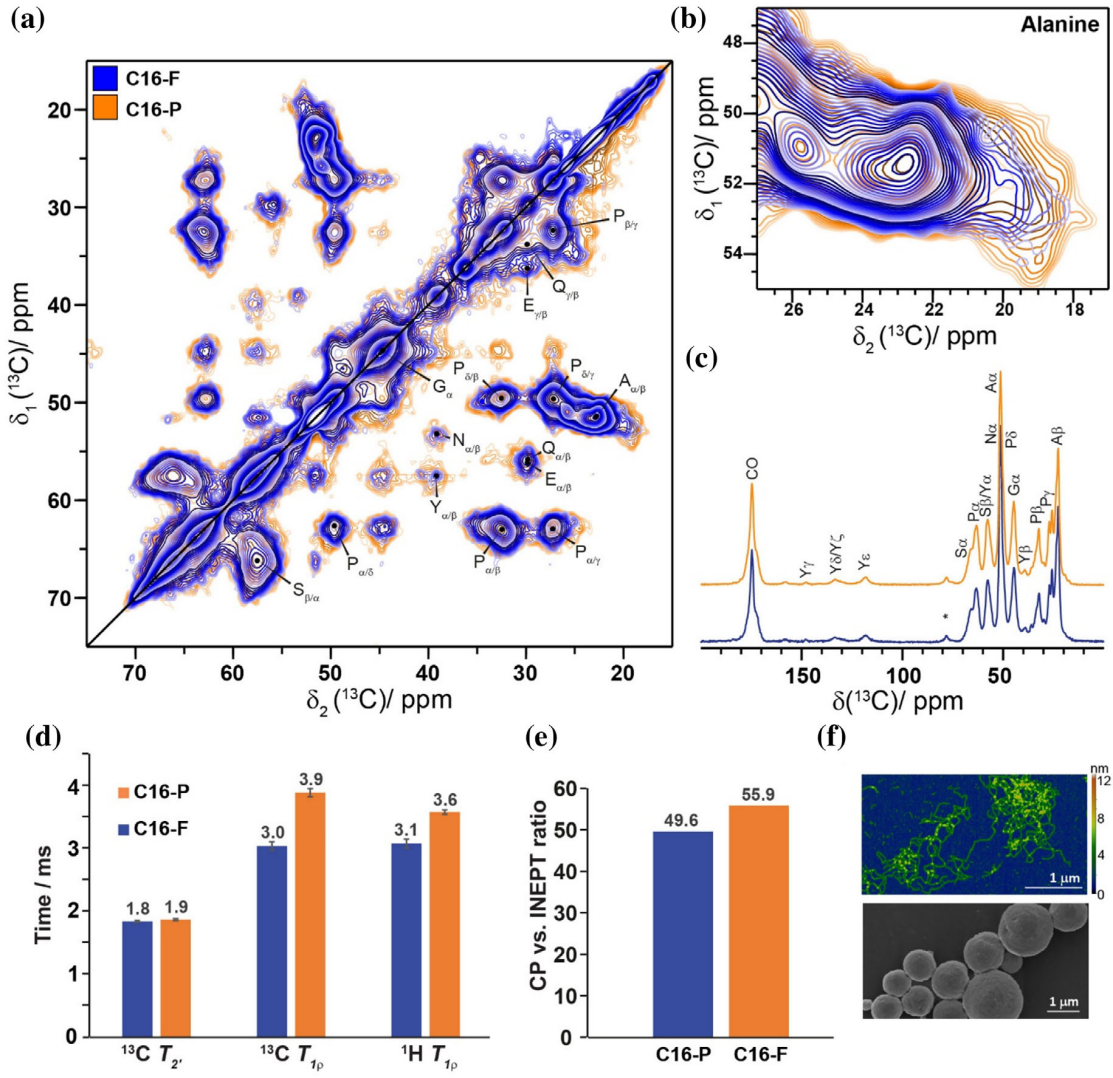
The particles and fibrils are macroscopically different, but have very similar NMR spectra. (a) Overlay of the ^13^C‐^13^C DARR spectra. (b) Zoom‐in of the Ala region of the DARR in (a). (c) Overlay of the ^1^H‐^13^C 1D CP‐MAS spectra. The spectra have been recorded at a magnetic‐field strength of 16.4 T and a MAS frequency of 17.0 kHz. (d) Boxplot with the ^13^C and ^1^H *T*_1ρ_ and ^13^C *T*_2_ bulk relaxation times. The error bars mark the standard deviation obtained from the fit of the relaxation data. (e) Boxplot with the ratio of the CP signal integral over the INEPT signal integral. Color code: Blue C16‐F, orange C16‐P. (f) AFM height image of C16‐F (top) and SEM image of C16‐P (bottom) samples. The samples have been taken from the MAS rotor after using those for several days in the MAS experiments. Color bar 0–13 nm.

Next, we investigated possible differences in terms of structure and dynamics in three obviously different morphologies (Slotta et al., 2008) namely the C16‐F, the C16‐F self‐assembled in 50 mM KPi and C16‐P samples. Interestingly, no significant differences have been observed in the 2D ^13^C‐^13^C DARR spectra (Figure 8a, Figure S9) as well as the ^13^C‐^13^C INEPT‐TOBSY spectra (Figures S3 and S10) probing the atomic level. Especially the Ala region, which forms the β‐sheet structure, is highly similar (Figure 8b) confirming previous observations of very similar β‐sheet content in infrared spectra (~40%) of eADF4(C16) nanofibrils and particles (Humenik et al., 2018). The only obvious difference between the spectra arises from the better signal‐to‐noise ratio of the sample with C16‐P, which can be attributed to a higher filling factor of the NMR rotor. Likewise, the 1D ^1^H‐^13^C CP spectra of the C16‐F and C16‐P samples show no significant differences (Figure 8c).

Since no spectral differences (i.e. chemical‐shift perturbations or relative intensity differences) could be detected pointing to structural differences between particles and nanofibrils, we investigated whether the dynamics of the two samples are also similar. Hence, we measured ^13^C *T*_1ρ_ and *T*_2_′, as well as ^1^H *T*_1ρ_ relaxation times (Figure 8d, for the individual data see Figure S11). The relaxation times presented here were obtained by integrating the bulk signal and fitting the data as a rough estimate to a mono‐exponential decay curve. With the relaxation times discussed herein, motions in the μs to ms regimes can be probed (Palmer & Massi, 2006; Schanda & Ernst, 2016). Those are typically slow, domain movements, loop motions or sidechain rotations in a protein. In Figure 8d, both ^1^H and ^13^C *T*_1ρ_ times are with 3.6 ± 0.05 ms and 3.9 ± 0.07 ms for C16‐F and C16‐P, respectively, slightly larger for the C16‐P sample compared to 3.0 ± 0.03 ms and 3.1 ± 0.07 ms, respectively, for the nanofibril sample. The slightly longer *T*_1ρ_ values indicate less motion in the C16‐P sample. ^1^H *T*_1ρ_ values previously reported for dragline silk of *Nephila edulis* and *Argiope keyserlingi* were one order of magnitude longer in the fibers in comparison to the samples studied herein (Kishore et al., 2002). The *T*_1ρ_ relaxation times for eADF4(C16) also differ significantly from that of films cast from a recombinant *Trichonephila clavipes* variant (Wu et al., 2024), which might be caused by differences in silk sample preparation leading for instance to different degrees of hydration and different degrees of crystallinity. The dynamics within a protein sample are also encoded in the polarization transfer efficiencies of CP and INEPT experiments as discussed above (Aebischer & Ernst, 2024; Topgaard, 2023). The higher the ratio of the CP versus the INEPT signal, the larger the rigid fraction of the sample. The calculated ratios for fibrils and particles are again rather similar with a slightly higher ratio for the nanofibrillar sample (Figure 8e). This small difference might arise from a larger amount of highly mobile components in the C16‐P sample. The appearance of the samples using AFM for C16‐F and SEM for C16‐P was probed after MAS to ensure that the different morphologies are not influenced by MAS (Figure 8f). And indeed, the imaging still reveals the expected features, for example, shows the integrity of the microparticles.

## 3 CONCLUSION

In this study, we gained insights into the structural and dynamic properties of the recombinant spider silk protein eADF4(C16) in its nanofibrillar and particulate morphology. Even though they are macroscopically different, no significant structural differences could be found at the atomic level nor in their dynamics. Their Ala and flanking Ser residues form an extended β‐sheet structure which is a mixture of rectangular and staggered β‐sheets. This formation of β‐sheets is a central process during self‐assembly. The Tyr sidechains are rigidified, which points to their engagement in π–π‐stacking interactions among each other. Also, Glu and Pro might have important roles therein. All Glu residues are deprotonated, which suggests that they are pointing to the outside of the fibrillar structure. The Pro residues appear in *trans*‐ as well as in *cis*‐conformation, which might be important for the control of the β‐sheet formation. The structural insights are supported by an AlphaFold3 model. All this information is crucial for understanding the self‐assembly process of recombinant eADF4(C16) structures. This understanding will enable us to design novel biomaterials with intended properties. The presented elucidation of the protein structure provides insights into the positioning and surface accessibility of amino‐acid residues within the self‐assembled architecture, offering clues to potential sites for chemical modification (e.g., highly exposed Glu residues) or strategies for genetic fusion on termini, allowing development of functionalized fibril‐based hydrogel systems for biosensing and tissue engineering (Herrmann et al., 2021; Humenik et al., 2018; Lechner et al., 2022; Malda et al., 2013; Ng et al., 2025). However, using structural information to rationally design new protein sequences with defined self‐assembly properties or predictable final morphologies remains challenging. The overall assembly process is governed by a delicate balance between assembly kinetics, subtle residue–residue interactions, and external conditions such as protein concentration and the presence of kosmotropic ions.

## 4 MATERIALS AND METHODS

### 4.1 Protein fermentation and purification

The fermentation procedures were adapted to obtain ^13^C‐ and ^15^N‐labeled protein variants. Fed‐batch fermentation was carried out in *Escherichia coli* BL21Gold (DE3) harboring pET29 vectors. Cultures were first grown in a complex medium for 12–14 h at 30°C until reaching an OD_600_ of ~30. Cells were harvested under sterile conditions by centrifugation at 5000 rpm for 20 min at room temperature. The resulting pellet was washed twice with 25 mM Tris/NaCl, pH 7.5, and resuspended in M9 minimal medium supplemented with 20 g L^−1 13^C‐glucose and 1.5 g L^−1^ (^15^NH_4_)_2_SO_4_. After induction with 0.1 mM IPTG, isotopically enriched protein was expressed for 14–16 h at 25°C. The protein purification was performed as described previously. Purification of the engineered protein eADF4(C16) followed a column‐free strategy (Huemmerich et al., 2004) and yielded a T7‐tagged construct: *(M)ASMTGGQQMGRGSM*(GSSAAAAAAAASGPGGYGPENQGPSGPGGYGPGGP)_16_G (T7)‐tag is italicized, Met1 (in parentheses) is post‐translationally cleaved (Hovanová, Hovan, Žoldák, et al., 2023; Humenik & Scheibel, 2014) The eADF4(C2) variant was expressed as a His‐SUMO‐tagged construct and purified via affinity chromatography (Humenik et al., 2014) resulting in the tag‐free protein SM(GSSAAAAAAAASGPGGYGPENQGPSGPG GYGPGGP)_2_G. The identity of the ^13^C/^15^N‐labeled proteins was confirmed using MALDI‐TOF (Figure S12) as described previously (Hovanová, Hovan, Žoldák, et al., 2023).

### 4.2 Protein solubilization and self‐assembly

Stock solutions of eADF4(C16) and eADF4(C2) were prepared by dissolving the lyophilized protein at a concentration of 4 mg/mL in 6 M guanidinium thiocyanate. The solution was subsequently dialyzed against 10 mM Tris–HCl buffer (pH 8.0) with four buffer exchanges: three after 2 h and one after 16 h. To obtain the monomeric form of the protein, the solution was subjected to ultracentrifugation at 195,000*g* for 60 min at 4°C using a Beckman Optima centrifuge, as described by Humenik et al. (2015a, 2015b). Protein concentration was determined using a NanoDrop spectrophotometer (PeqLab, VWR, Germany).

Particle formation of the C16‐P sample was carried out under conditions established in our previous studies (Lammel et al., 2008; Slotta et al., 2008), which demonstrated that sphere characteristics, such as size, size distribution, and cluster formation, depend strongly on the preparation method, including dialysis, simple pipette mixing, or micromixing using a T‐mixing element. In the present study, particle formation was achieved using the simplest yet effective approach—pipette‐mixing. The protein was precipitated at a concentration of 0.5 mg/mL by rapid pipette mixing with cyclic aspiration of an equal volume of 2 M potassium phosphate (KPi) buffer at pH 8.0. The observed particle size and distribution (Figure 8e) closely matched those reported in our previous study (Lammel et al., 2008). Nanofibril assembly (C16‐F samples) was achieved by incubating the protein in 150 mM or 50 mM KPi, pH 8.0, for 48 h. The shorter variant eADF4(C2) was assembled in 150 mM KPi, pH 8.0 for 5 days. Prior to NMR analysis, both samples were concentrated to 20 mg/mL by centrifugation at 3000 *g* using a benchtop centrifuge.

### 4.3 Morphology of particles and nanofibrils

The C16‐P and C16‐F samples were examined before and after NMR analysis and showed no detectable morphological changes.

For nanofibril imaging, we employed atomic force microscopy (AFM). Scanning electron microscopy (SEM) and transmission electron microscopy (TEM) are less suitable for visualizing these nanofibrils because of the extensive sample preparation required, particularly metal sputter coating for SEM and heavy‐metal staining for TEM, which can introduce imaging artifacts and distort native nanostructures. In contrast, AFM requires no additives. Similarly to previous studies (Hovanová, Hovan, Žoldák, et al., 2023; Humenik et al., 2014; Humenik & Scheibel, 2014), the fibrils were resuspended at 0.2 mg/mL in Milli‐Q water, and 50 μL of the suspension was deposited onto a freshly cleaved mica slide for 5 min. The adsorbed fibrils were washed four times with 50 μL of Milli‐Q water using blotting with fiber‐free paper. AFM was performed in dynamic mode using a Nanosurf Flex system (Nanosurf AG, Liestal, Switzerland) equipped with a C3000i controller and a Tap190Al‐G cantilever (BudgetSensors; force constant: 48 N/m, resonance frequency: 190 kHz). Image analysis was conducted using Gwyddion software (version 2.65).

Due to their micrometer‐scale size, the particles are not suitable for visualization using AFM, but can be conveniently imaged using SEM. Hence, according to previous studies (Lammel et al., 2008; Slotta et al., 2008), particle samples were resuspended at 1 mg/mL in Milli‐Q water, and 50 μL was deposited onto Nunc™ Thermanox™ slides (Thermo Fisher Scientific, Waltham, MA, USA). After adsorption, the slides were washed four times with 50 μL of Milli‐Q water and mounted onto SEM stubs (Plano GmbH, Germany) using aluminum tape. The samples were sputter‐coated with a 2 nm platinum layer using a Leica EM ACE 600 sputter coater (Leica, Germany) and visualized by SEM using a Thermo Scientific (FEI) Apreo VS system equipped with a field emission gun and SE2 detector, operating at 2 kV (Thermo Fisher Scientific, Germany).

### 4.4 Solid‐state NMR spectroscopy

The uniformly ^13^C‐/^15^N‐labeled eADF4(C16) nanofibrils and particles were transferred in 3.2 mm solid‐state NMR rotors by means of ultracentrifugation (14.300 *g*, 4°C, ~18 h).

Solid‐state NMR spectra were recorded at a MAS frequency of 17.0 kHz and at 11.7 and 16.4 T static magnetic‐field strengths in 3.2 mm Bruker standard triple‐resonance and “Efree” probes. The spectral acquisition details are reported in Table S1.

2D NCαCX (X = Pro) spectra have been recorded based on a standard 2D NCαCX correlation sequence by setting the carrier frequency for the specific CP step (Baldus et al., 1998) to the ^13^Cα of the Pro residues.

The ^13^C *T*_2_’ relaxation time experiments have been recorded at 17.0 kHz and 11.7 T, using a standard CP based Hahn echo sequence and varying the length of the echo delay between 1 μs and 40 ms. For the delays used we refer to Figure S10a. The ^1^H and ^13^C *T*_1ρ_ relaxation experiments have been recorded at 17.0 kHz and 16.4 T. A 45‐kHz spin‐lock pulse on both protons and carbons, in the respective experiments, has been employed in order to avoid rotary resonance conditions. In this case, the length of the spin‐lock pulse has been varied for both experiments between 200 μs and 40 ms, and we refer to Figure S10b,c for the delays used. Relaxation data have been processed and fitted using an exponential decay function with the standard routine implemented in Topspin (version 4.1.4, Bruker Biospin).

Spectra were processed with the software Topspin (versions 3.6.4, 4.1.3, and 4.1.4, Bruker Biospin). The 2D spectra were processed with a shifted (SSB = 2.5) square sine apodization function and cut in the direct dimension to a total acquisition time of 10.2 ms, and additionally in the indirect dimension to 7.6 ms only for the 2D DARR experiments. 2D NCαCX spectra were processed with an SSB = 2 and cut in the indirect dimension to 3.8 ms. An automated baseline correction in the indirect and direct dimensions was applied where needed. Zero filling to up to double the number of points was applied for all spectra.

2D spectra were analyzed with the software CcpNmr (v.2.4.2) (Fogh et al., 2002). All spectra were referenced to 2,2‐dimethyl‐2‐silapentane‐5‐sulfonate (DSS) according to IUPAC using the ^1^H NMR resonance (0.00 ppm). ^13^C and ^15^N chemical‐shift referencing was achieved by employing the reported frequency ratios (Harris et al., 2001).

To obtain the CP versus INEPT ratios the spectra were integrated with the software ssNake v1.5 (van Meerten et al., 2019).

### 4.5 Alpha‐Fold modeling

The structure of eADF4(C16) was predicted using AlphaFold3 (Abramson et al., 2024) with its default settings and template mode. To investigate the influence of the number of repeat units on the confidence of structure prediction, the eADF4 structure was predicted for different repeat numbers ranging from C2 to C16.

## AUTHOR CONTRIBUTIONS

**Nina Wehr:** Investigation; writing – original draft; formal analysis; visualization; data curation. **Ettore Bartalucci:** Investigation; writing – original draft; visualization; formal analysis; data curation. **Sabrina Smid:** Writing – review and editing; investigation; formal analysis. **Georg Künze:** Writing – review and editing; investigation; visualization; formal analysis; data curation. **Martin Humenik:** Conceptualization; writing – review and editing; supervision. **Thomas Scheibel:** Conceptualization; writing – review and editing; supervision. **Thomas Wiegand:** Conceptualization; writing – original draft; supervision; funding acquisition.

## Supporting information


**Data S1.** Supporting Information.

## Data Availability

The scripts used in this manuscript are available through a public Github repository hosted at: https://github.com/ebartalucci/eADF4C16.git. The spectra and AlphaFold3 models have been deposited on the Zenodo archive (access code: 10.5281/zenodo.17692129).

## Footnotes

* The total ^13^C linewidths (Δ^total^) is given by the sum of the homogeneous (Δ^homo^) and inhomogeneous broadening (Δ^inhomo^). The *T*_*2*_ *‘* transverse relaxation times (following definitions as reported in ref. Penzel et al., [2019]) obtained from the spin‐echo decay curves have been determined to 1.0 ± 0.04 ms and 1.1 ± 0.04 ms for C16‐F and C16‐P, respectively. The homogeneous contribution to the ^13^C linewidth (1/[π*T*_2_′]) would therefore be around 320 Hz and 290 Hz for C16‐F and C16‐P, respectively. Hence, the total observed FWHM linewidth is dominated by inhomogeneous broadening effects.
