# Supplementary material for: Structural and dynamic similarities of nanofibrils and microparticles of engineered spider silk proteins probed by solid‐state NMR spectroscopy
Source: Protein Sci. 2026 Jan 20;35(2):e70460. doi: 10.1002/pro.70460 (PMC12817280; doi:10.1002/pro.70460)
Supplement: Supplementary file 1 — Data S1. Supporting Information. [file PRO-35-e70460-s001.docx]

**Supporting Information for:**

**Structural and Dynamic Similarities of Nanofibrils and Microparticles of Engineered Spider Silk Proteins probed by Solid-State NMR Spectroscopy**

Nina Wehr^a,#^, Ettore Bartalucci^a,b,#^, Sabrina Smid^a^, Georg Künze^c,d,e^, Martin Humenik^f^, Thomas Scheibel^f-k,*^, and Thomas Wiegand^a,b,*^

^a^ Institute of Technical and Macromolecular Chemistry, RWTH Aachen University, Worringerweg 2, 52074 Aachen, Germany

^b^ Max Planck Institute for Chemical Energy Conversion, Stiftstr. 34-36, 45470 Mülheim/Ruhr, Germany

^c^ Institute for Drug Discovery, Medical Faculty, Leipzig University, 04103 Leipzig, Germany

^d^ Interdisciplinary Center for Bioinformatics, Leipzig University, 04107 Leipzig, Germany

^e^ Center for Scalable Data Analytics and Artificial Intelligence, Leipzig University, 04105 Leipzig, Germany

^f^ Department of Biomaterials, University Bayreuth, 95447, Bayreuth, Germany.

^g^ Center of Material Science and Engineering (BayMat), University Bayreuth, 95447, Bayreuth, Germany.

^h^ Bavarian Polymer Institute (BPI), University Bayreuth, 95447, Bayreuth, Germany.

^i^ Center of Colloids and Interfaces (BZKG), University Bayreuth, 95447, Bayreuth, Germany.

^j^ Bayreuth Center for Molecular Biosciences (BZMB), University Bayreuth, 95447, Bayreuth, Germany.

^k^ North Bavarian NMR-Center, University Bayreuth, 95440, Bayreuth, Germany.

#: Both authors contributed equally.

*: Corresponding authors. T.S.: Thomas.Scheibel@uni-bayreuth.de, T.W.: thomas.wiegand@cec.mpg.de

**Supplementary Figures**


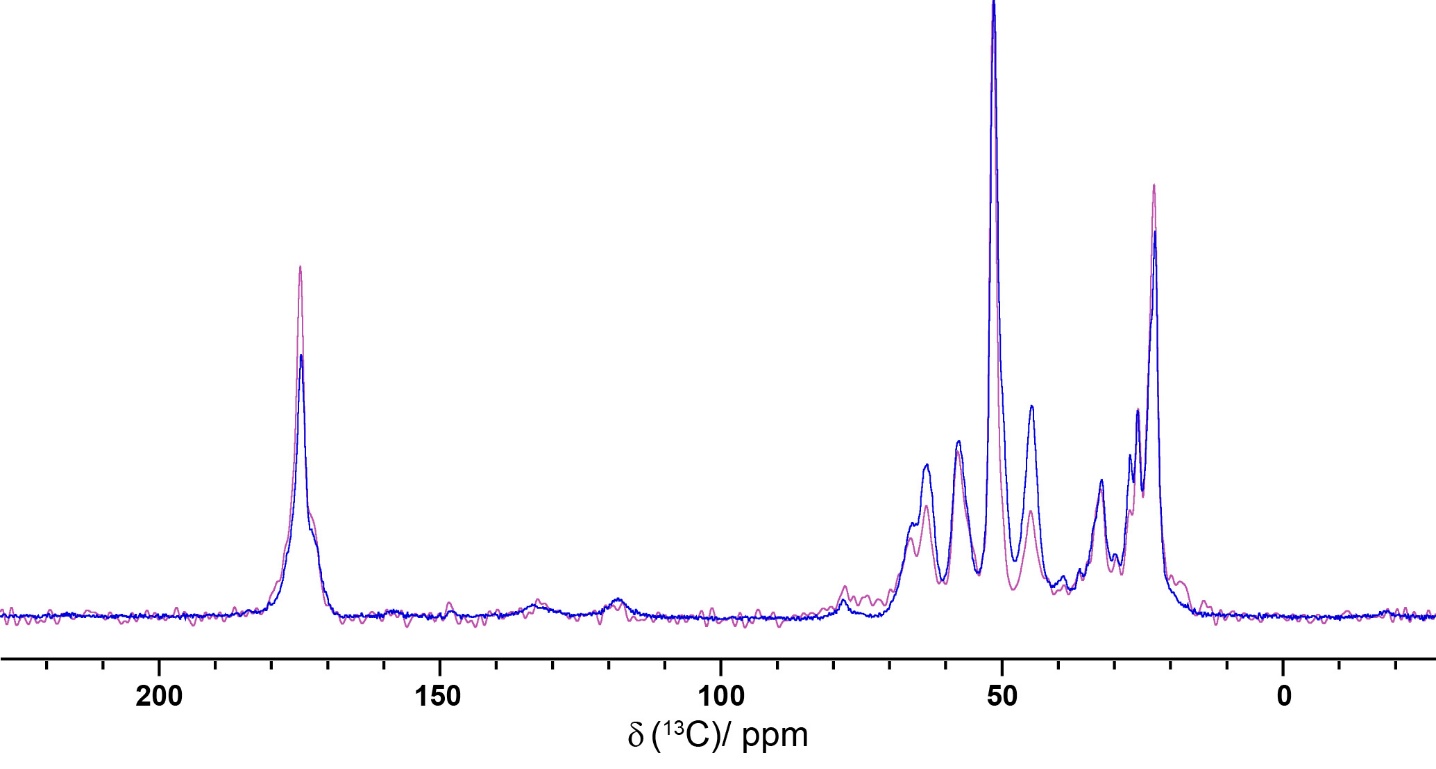
 **Figure S1:** *The ^13^C linewidths of the resonances in the ^13^C CP-MAS spectra do not change for a shorter protein construct.* ^13^C CP-MAS spectra of C16-F (blue) and C2-F (purple). The same resonances are appearing in both spectra, and the linewidths are rather similar for both constructs. Some resonances differ in intensity, e.g. as a consequence of different polarization transfer efficiencies.


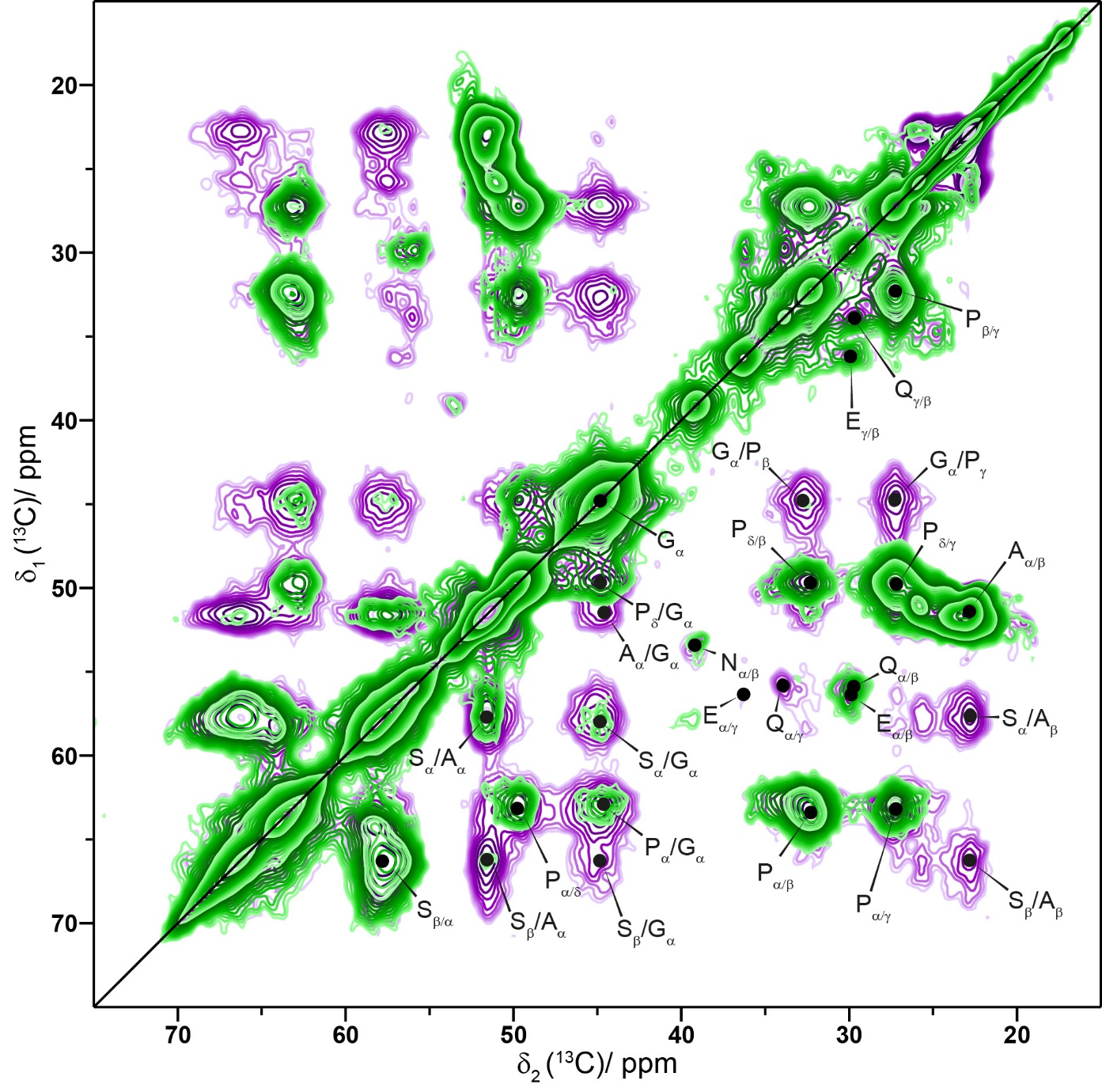
**Figure S2:** *The 200 ms DARR spectrum of C16-F self-assembled with 50 mM KP_i_ probes further long-range contacts between Ala-Ser, Ser-Gly, Ala-Gly and Pro-Gly residues.* ^13^C-^13^C 200 ms DARR spectrum (violett) and the ^13^C-^13^C 20 ms DARR spectrum (green) of the C16-F self-assembled with 50 mM KP_i_. The aliphatic region and some resonance assignments are shown in the spectrum.


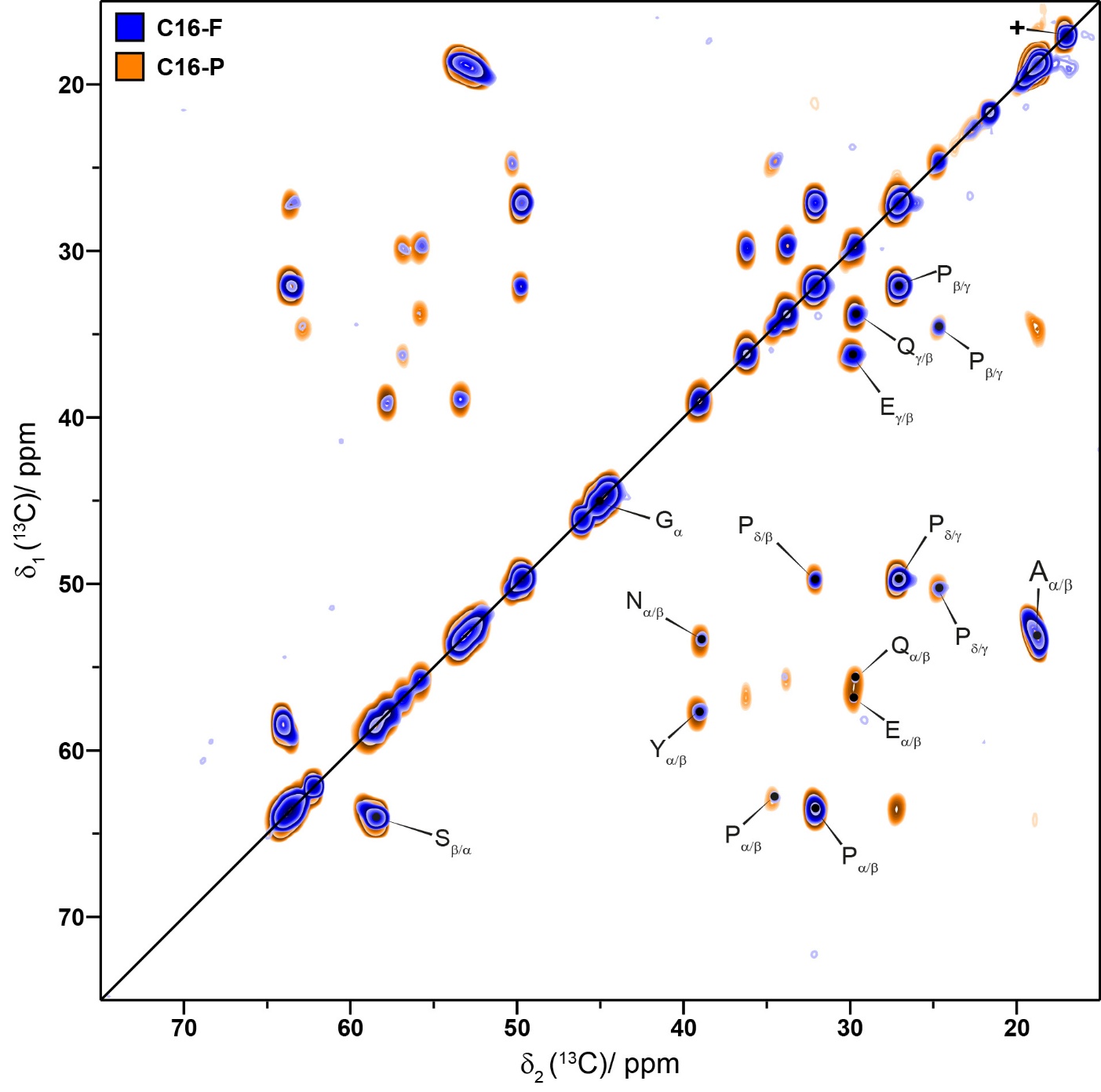
 **Figure S3:** *The INEPT-TOBSY spectra of C16-F and C16-P are very similar.* Zoom into the aliphatic spectral region of ^13^C-^13^C INEPT-TOBSY spectra of C16-F (blue) and C16-P (orange). The resonance marked with a + on the diagonal is assigned to Met residues, which are part of the tag used (amino acid sequence MASMT GGQMG RGSM).


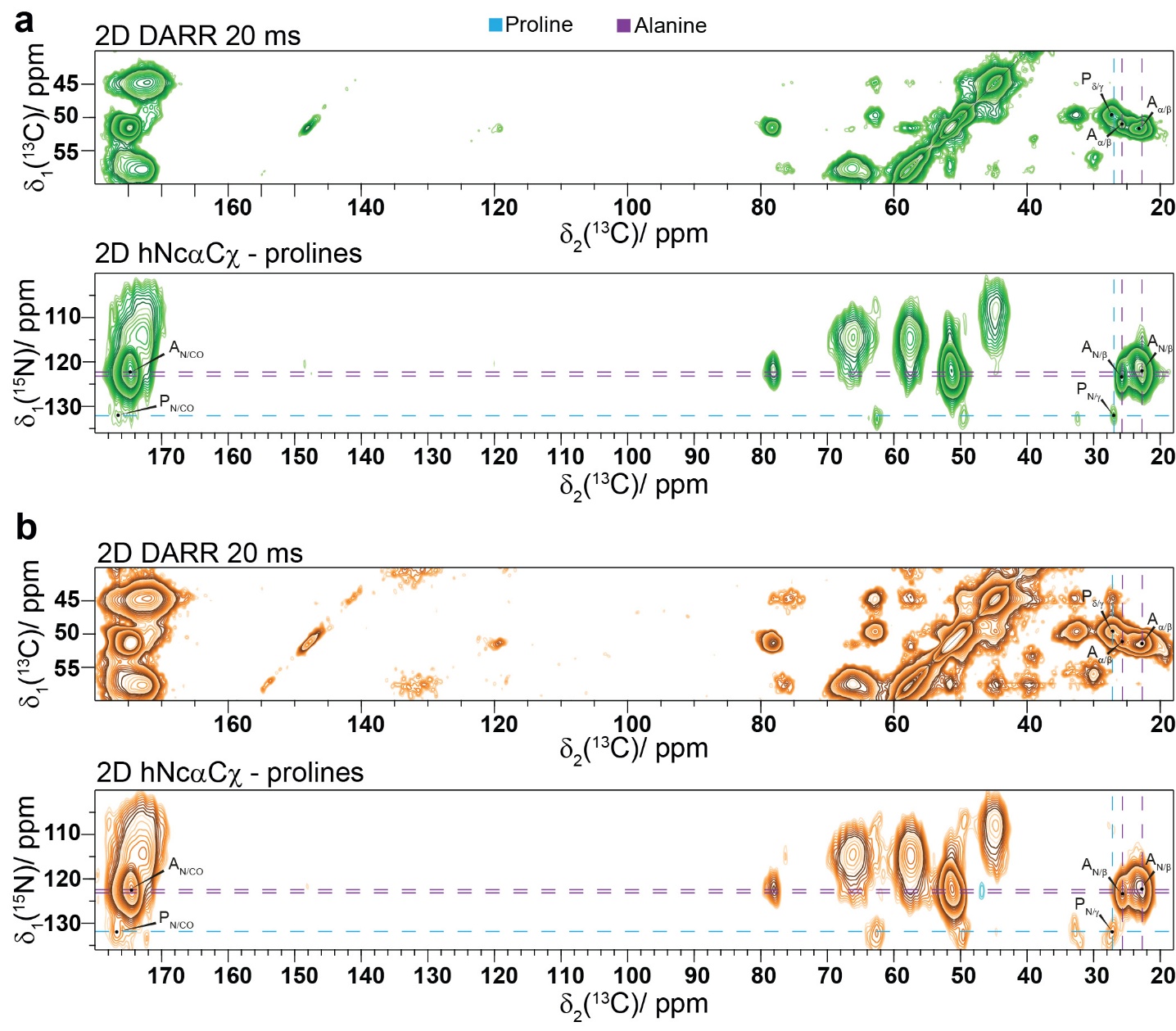


**Figure S4:** *The 2D hNcaCx spectra allow distinguishing proline from alanine resonances.* (**a**) The top shows a zoom into the 2D ^13^C‑^13^C 20 ms DARR spectrum of C16-F self-assembled with 50 mM KP_i_. The bottom shows the 2D ^15^N-^13^C hNcαCχ spectrum on the same sample, which enables the distinction of Ala and Pro residues in the nitrogen dimension due to the typically more downfield shifted ^15^N resonance of Pro. The blue dashed lines mark the Pro resonances and the purple dashed line the Ala resonances. (**b**) The top shows a zoom into the 2D ^13^C-^13^C 20 ms DARR spectrum of C16-P. The bottom shows the 2D ^15^N-^13^C hNcαCχ experiment on the same sample. The distinction between Ala and Pro residues is made here as described for (**a**).


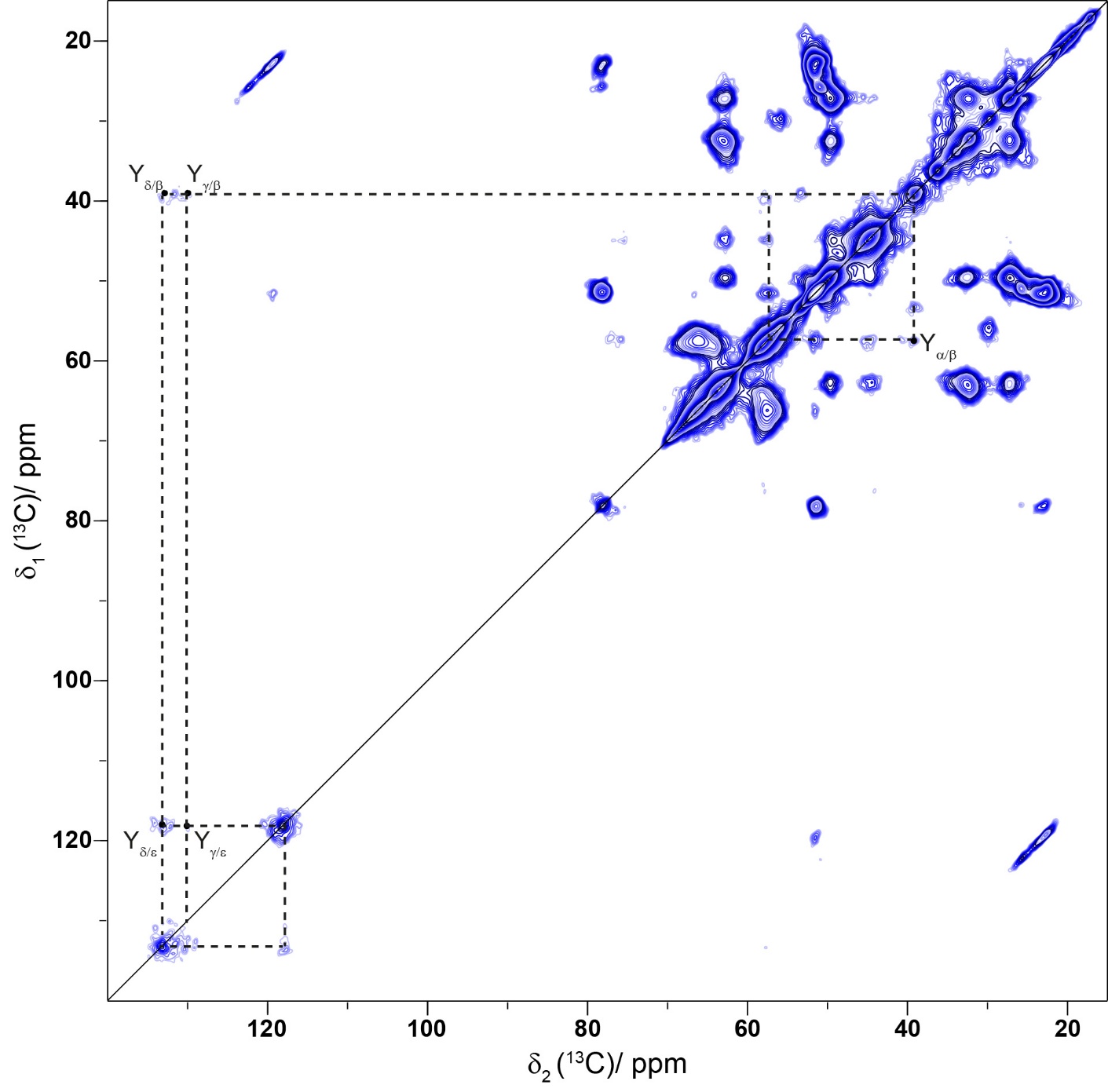


**Figure S5:** *The Tyrosine spin system shows only intraresidual connections.* 2D ^13^C-^13^C DARR spectrum of C16-F, showing the aliphatic region as well as the aromatic region of the Tyr sidechain. The Tyr cross peaks are labelled and the spin system is marked with a dashed line.


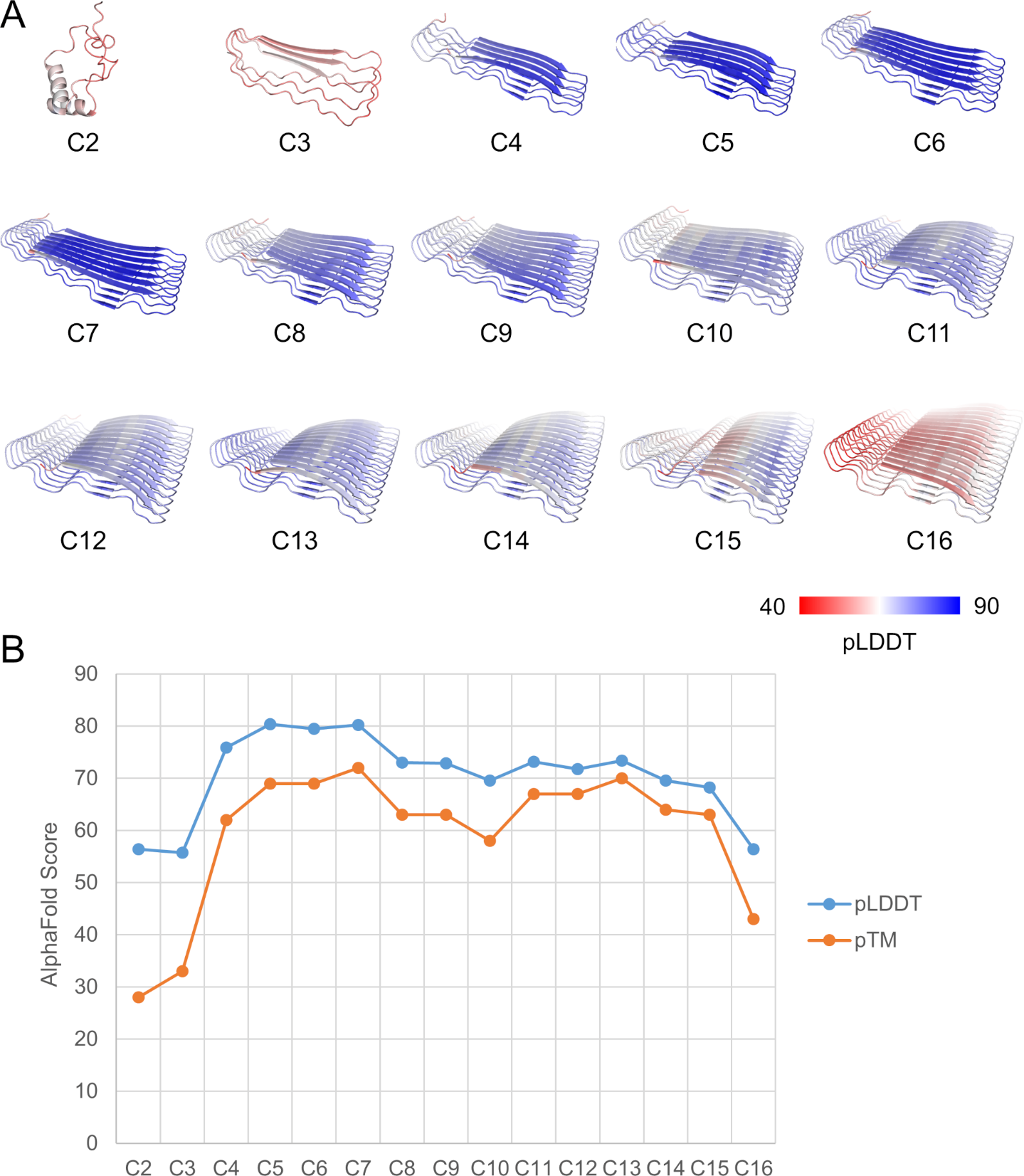


**Figure S6:** *The AlphaFold3 models of various eADF4 constructs ranging from C2 to C16.* (**a**) AlphaFold3 models of different eADF4 constructs, ranging from C2 to C16. The confidence of the model is based on the pLDDT score as the color of the model ranging from a low pLDDT score in red to a high pLDDT score in blue. The constructs C4 to C7 show a high confidence. (**b**) pLDDT score (blue) and the pTM score (orange) for each modeled construct.


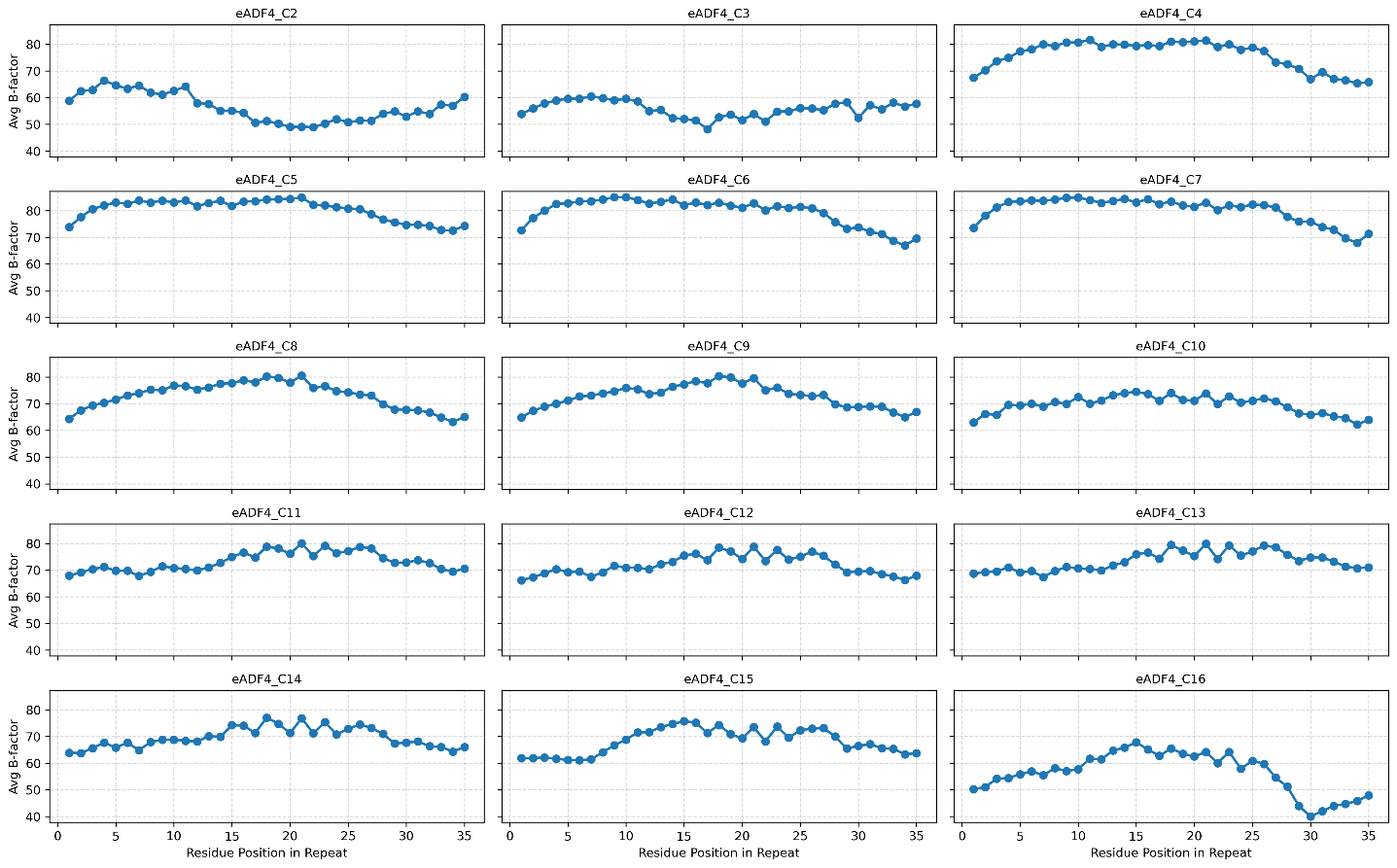


**Figure S7:** Cα-atom pLDDT scores for each position in the 35-residue eADF4 repeat, averaged across all repeats for each construct (C2 to C16). The plot illustrates residue-level confidence variations in the AlphaFold3 predictions for the different eADF4 molecules.


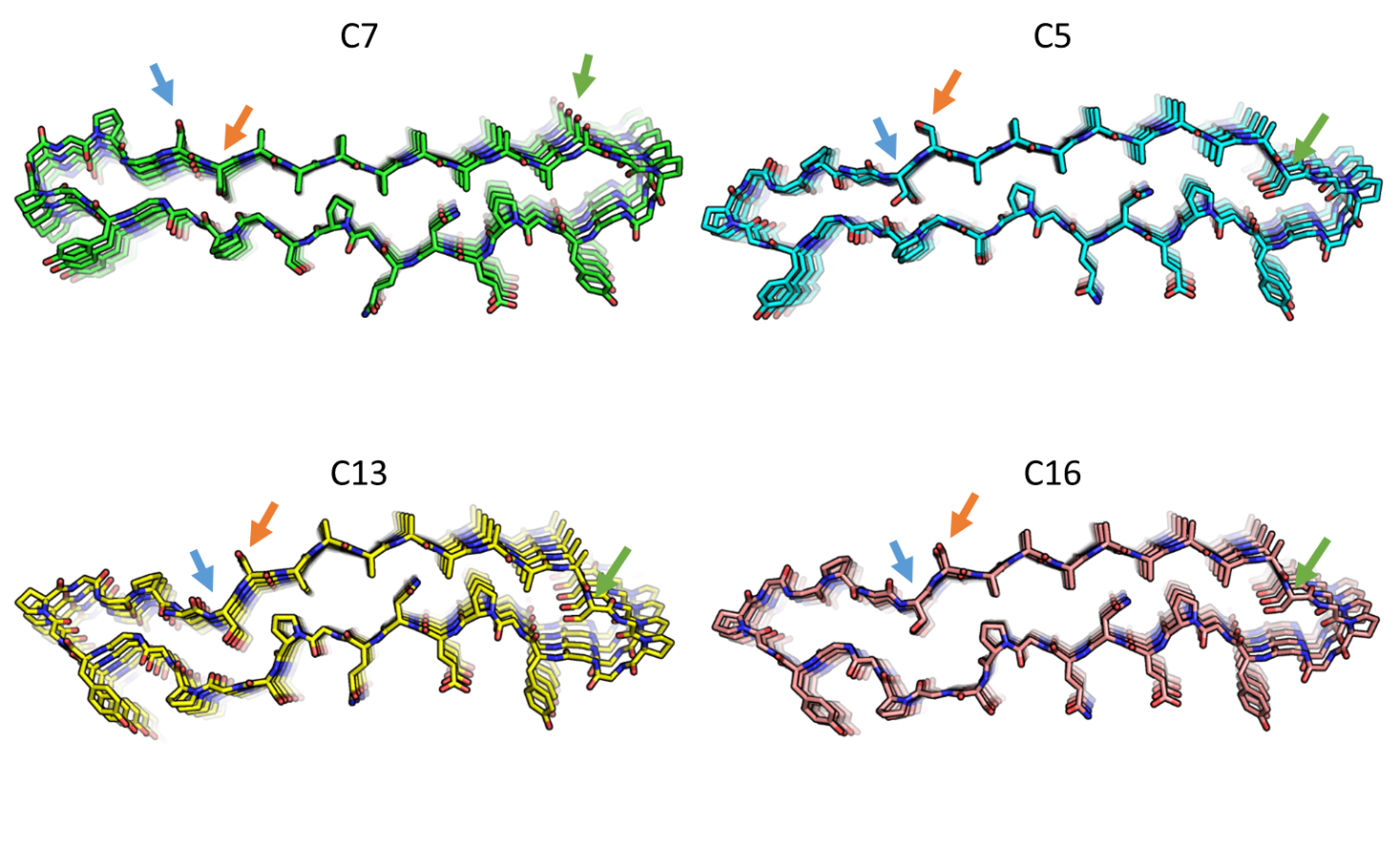
 **Figure S8:** *The AlphaFold3 models of the different eADF4 constructs reveal only small structural differences.* AlphaFold3 models of the eADF4(C7) construct (green, top left), the eADF4(C5) construct (blue, top right), the eADF4(C13) construct (yellow, bottom left) and the eADF4(C16) construct (pink, bottom right). The C5 model has the same configuration as the longer constructs such as C13 and C16. Three serine residues for which different sidechain orientations in the different models have been observed are highlighted by colored arrows.


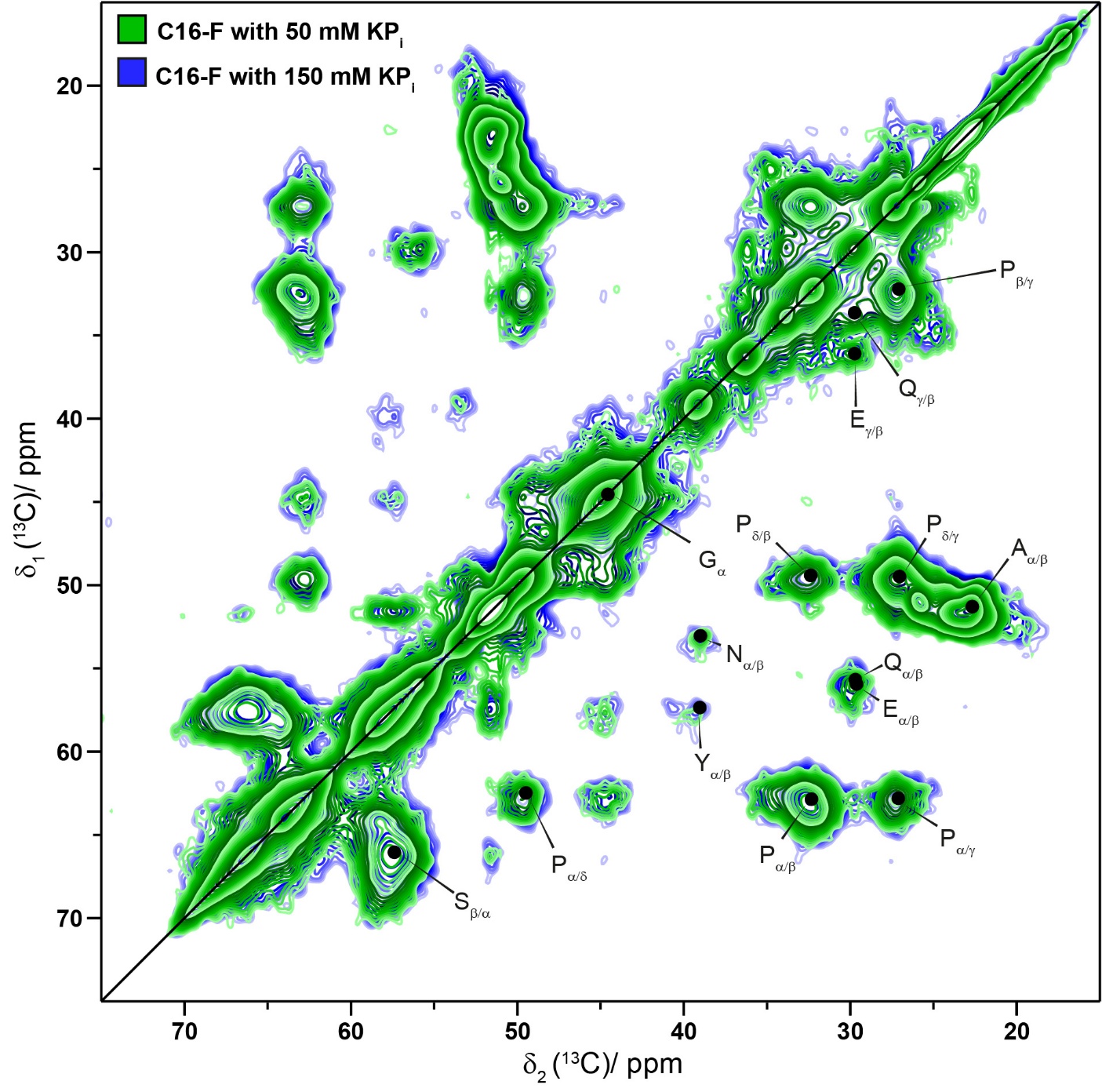
**Figure S9:** *The 20 ms DARR spectra of C16-F and C16-F self-assembled with 50 mM KPi are very similar.* Overlay of ^13^C-^13^C DARR spectra of C16-F (blue) and C16-F self-assembled with 50 mM KP_i_ (green) showing the aliphatic region.


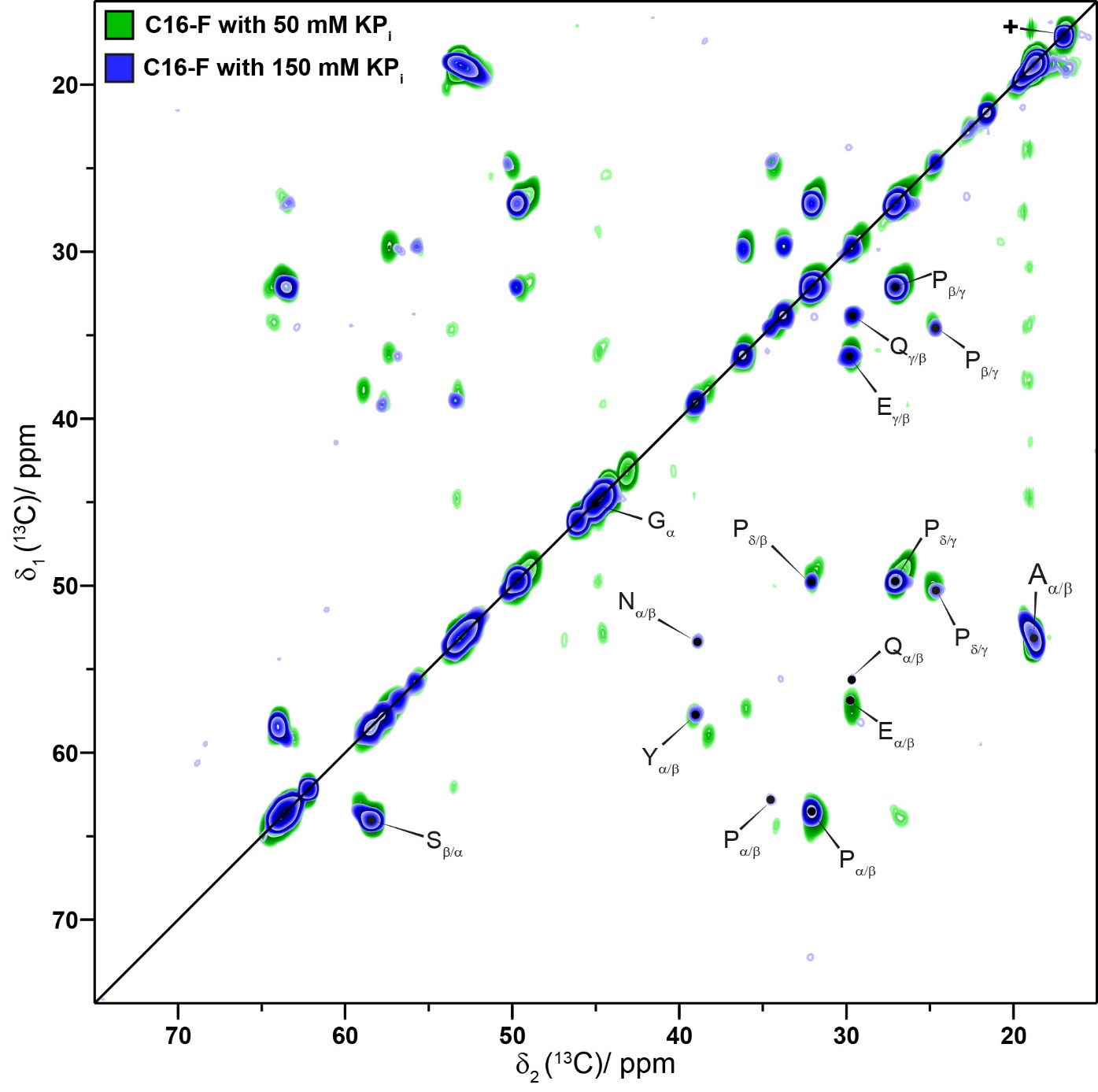
**Figure S10:** *The INEPT-TOBSY spectra of C16-F and C16-F self-assembled with 50 mM KP_i_ are very similar.* ^13^C-^13^C INEPT-TOBSY spectrum of C16‑F (blue) and C16‑F self-assembled with 50 mM KPi (green) showing the aliphatic region. The resonance marked with a + on the diagonal are assigned to Met residues, which are part of the used tag composed of MASMT GGQMG RGSM.


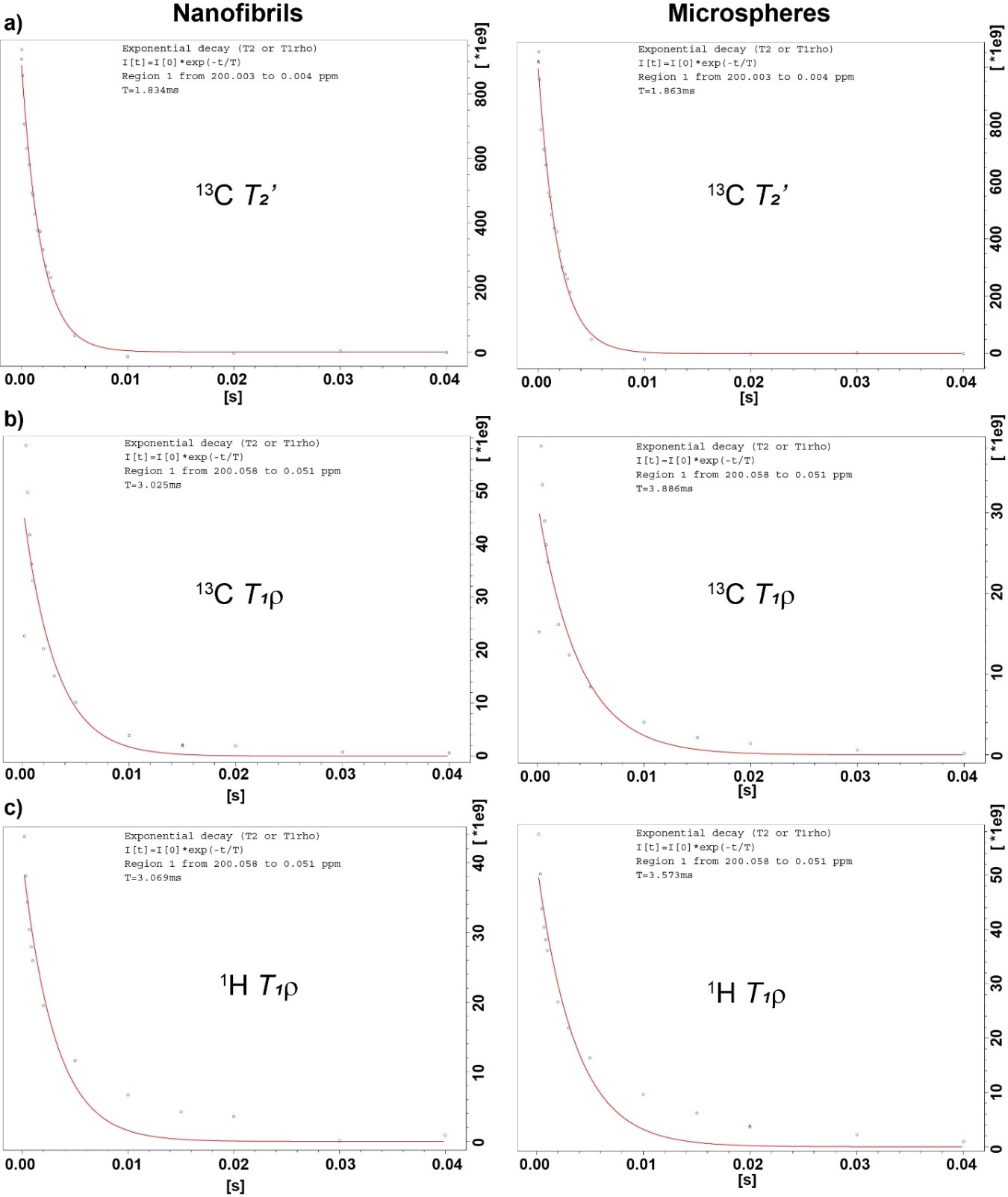
**Figure S11:** *Monoexponential fits for the determination of nuclear relaxation times reported in Figure 7.* (**a**) ^13^C spin-echo decay curves and monoexponential fits (in red) to determine the bulk *T_2_’* relaxation time of C16-F (left) and C16-P (right). (**b**) Integration of ^13^C spin-lock spectra to determine bulk *T_1ρ_* relaxation times of C16-F (left) and C16-P (right). The red curve shows a monoexponential fit. (**c**) Integration of ^1^H spin-lock spectra to determine bulk *T_1ρ_* relaxation times of C16-F (left) and C16-P (right). The red curve shows a monoexponential fit.


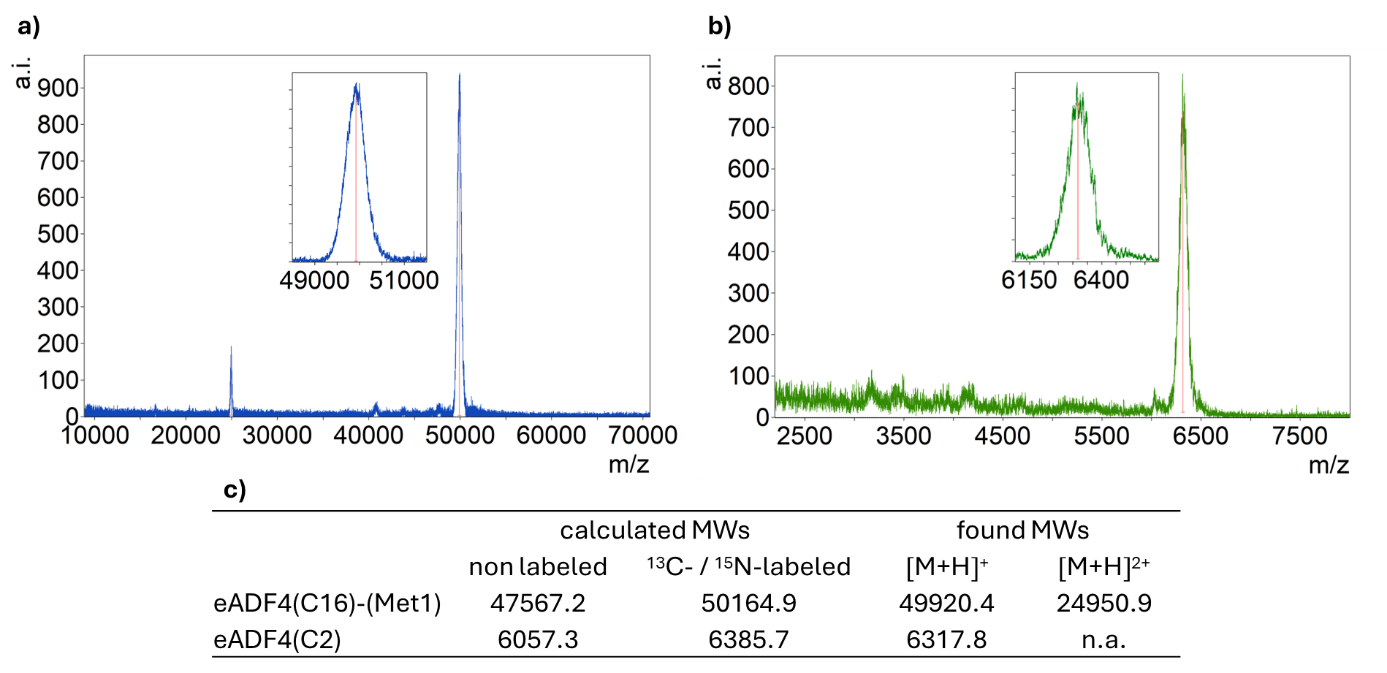


**Figure S12.** *MALDI-TOF spectra of the purified ^13^C/^15^N-labeled proteins:* eADF4(C16) in a), eADF4(C2) in b) and summary of calculated and found molecular masses as indicated. Taking into account the measured molecular weight and the molecular formula C₂₀₀₆H₂₉₂₆N₆₁₂O₇₄₁S₃ of eADF4(C16)-(Met1), the labeling grade can be estimated as follows: ¹³C fraction ≈ 1934/2006 ≈ 96%, ¹⁵N fraction ≈ 437/612 ≈ 71%. For eADF4(C2) with the molecular formula C₂₅₄H₃₇₃N₇₇O₉₅S₁, the labeling grade can be estimated as: ¹³C fraction ≈ 185/254 ≈ 73%, ¹⁵N fraction ≈ 77/77 = 100%. These values represent the most probable isotopic incorporation levels derived from the experimentally observed molecular masses.

**Table S1**: Overview about experimental parameters of the performed solid-state NMR experiments. For more details about the used adiabatic CP steps and the tangential shapes used see reference^1^.

| **Sample** | C16-F | C16-P | C16-F | C16-F |
| --- | --- | --- | --- | --- |
| **Experiment** | ^1^H-^13^C CP-MAS | ^1^H-^13^C CP-MAS | ^1^H-^13^C INEPT | ^13^C-^13^C DARR 20 ms |
| **Figure** | 1, 7 | 7 | 2 | 1,3,4,5,6,7 |
| ν_r_ / kHz | 17.0 | 17.0 | 17.0 | 17.0 |
| *B*_0_/ T | 16.4 | 16.4 | 16.4 | 16.4 |
| VTU / K | 272 | 272 | 272 | 272 |
| Probe | 3.2mm triple resonance | 3.2mm triple resonance | 3.2mm triple resonance | 3.2mm triple resonance |
| transfer I | ^1^H-^13^C CP | ^1^H-^13^C CP | ^1^H-^13^C INEPT for *J* = 240 Hz | ^1^H-^13^C CP |
| *ν*_1_(^1^H)/ kHz | 58.4 | 58.4 | - | 60.0 |
| *ν*_1_(^13^C)/ kHz | 43.9 | 43.9 | - | 44.7 |
| Shape | Tangent ^1^H | Tangent ^1^H | - | Tangent ^1^H |
| time / ms | 0.7 | 0.7 | - | 0.7 |
| transfer II | - | - | - | DARR |
| *ν*_1_(^1^H)/ kHz | - | - | - | 17 |
| time / ms | - | - | - | 20 |
| *t_1_* increments | - | - | - | 2048 |
| spectral width (*t_1_*) / kHz | - | - | - | 100 |
| acquisition time (*t_1_*) / ms | - | - | - | 10.24 |
| *t_2_* increments | 3072 | 3072 | 8192 | 3072 |
| spectral width (*t_2_*) / kHz | 100 | 100 | 100 | 100 |
| acquisition time (*t_2_*) / ms | 15.4 | 15.4 | 41.0 | 15.4 |
| Decoupling sequence | Spinal64 | Spinal64 | Waltz64 | Spinal64 |
| ^1^H decoupling power / kHz | 90 | 90 | 5 | 90 |
| interscan delay / s | 2.7 | 2.7 | 1.5 | 2.7 |
| number of scans | 128 | 128 | 128 | 4 |

**Table S1 continued**

| **Sample** | C16-P | C16-F (50 mM KP_i_) | C16-F | C16-P |
| --- | --- | --- | --- | --- |
| **Experiment** | ^13^C-^13^C DARR 20 ms | ^13^C-^13^C DARR 20 ms | ^13^C-^13^C INEPT-TOBSY | ^13^C-^13^C INEPT-TOBSY |
| **Figure** | 7, S4 | S9, S4, S2 | 2, 3, 5, 6, S5, S9, S10 | S9 |
| ν_r_ / kHz | 17.0 | 17.0 | 17.0 | 17.0 |
| *B*_0_/ T | 16.4 | 16.4 | 16.4 | 16.4 |
| VTU / K | 272 | 272 | 272 | 272 |
| Probe | 3.2mm triple resonance | 3.2mm triple resonance | 3.2mm triple resonance | 3.2mm triple resonance |
| transfer I | ^1^H-^13^C CP | ^1^H-^13^C CP | ^1^H-^13^C INEPT for *J* = 240 Hz | ^1^H-^13^C INEPT for *J* = 240 Hz |
| *ν*_1_(^1^H) / kHz | 60.0 | 51.0 | - | - |
| *ν*_1_(^13^C) / kHz | 46.2 | 35.7 | - | - |
| Shape | Tangent ^1^H | Tangent ^1^H | - | - |
| time / ms | 0.7 | 0.7 | - | - |
| transfer II | DARR | DARR | - | - |
| *ν*_1_(^1^H) / kHz | 17 | 17 | - | - |
| time / ms | 20 | 20 | - | - |
| *t_1_* increments | 2048 | 2560 | 455 | 455 |
| spectral width (*t_1_*) / kHz | 100 | 100 | 50 | 50 |
| acquisition time (*t_1_*) / ms | 10.3 | 12.8 | 4.6 | 4.6 |
| *t_2_* increments | 2048 | 3072 | 8192 | 8192 |
| spectral width (*t_2_*) / kHz | 100 | 100 | 100 | 100 |
| acquisition time (*t_2_*) / ms | 10.3 | 15.4 | 41.0 | 41.0 |
| Decoupling sequence | Spinal64 | Spinal64 | Waltz64 | Waltz64 |
| ^1^H decoupling power / kHz | 90 | 90 | 5 | 5 |
| interscan delay / s | 2.7 | 2.7 | 2.0 | 2.0 |
| number of scans | 4 | 8 | 64 | 64 |

**Table S1 continued**

| **Sample** | C16-F (50 mM KP_i_) | C16-F (50 mM KP_i_) | C16-P | C16-F (50 mM KP_i_) |
| --- | --- | --- | --- | --- |
| **Experiment** | ^13^C-^13^C INEPT-TOBSY | ^13^C-^13^C DARR 200 ms | ^15^N-^13^C hNCαCχ | ^15^N-^13^C hNCαCχ |
| **Figure** | S10 | S2 | S4 | S4 |
| ν_r_ / kHz | 17.0 | 17.0 | 17.0 | 17.0 |
| *B*_0_/ T | 16.4 | 16.4 | 16.4 | 16.4 |
| VTU / K | 272 | 272 | 255 | 255 |
| Probe | 3.2mm triple resonance | 3.2mm triple resonance | 3.2mm triple resonance “E-free” | 3.2mm triple resonance “E-free” |
| transfer I | ^1^H-^13^C INEPT for *J* = 240 Hz | ^1^H-^13^C CP | ^1^H-^15^N CP | ^1^H-^15^N CP |
| *ν*_1_(^1^H) / kHz | - | 51.0 | 60.0 | 60.0 |
| *ν*_1_(^13^C) / kHz | - | 35.7 | 46.0 | 46.0 |
| Shape | - | Tangent ^1^H | Tangent ^1^H | Tangent ^1^H |
| time / ms | - | 0.7 | 1 | 1 |
| transfer II | - | DARR | ^15^N-^13^C CP | ^15^N-^13^C CP |
| *ν*_1_(^1^H) / kHz | - | 17 | - | - |
| *ν*_1_(^15^N) / kHz | - | - | 10 | 10 |
| *ν*_1_(^13^C) / kHz | - | - | 13 | 12 |
| Shape | - | - | Tangent ^13^C | Tangent ^13^C |
| time / ms | - | 200 | 5 | 5 |
| transfer III | - | - | DARR | DARR |
| *ν*_1_(^1^H) / kHz | - | - | 17 | 17 |
| time / ms | - | - | 50 | 50 |
| *t_1_* increments | 455 | 2048 | 1160 | 1160 |
| spectral width (*t_1_*) / kHz | 50 | 100 | 66.7 | 66.7 |
| acquisition time (*t_1_*) / ms | 4.6 | 10.3 | 8.7 | 8.7 |
| *t_2_* increments | 8192 | 3072 | 2048 | 2048 |
| spectral width (*t_2_*) / kHz | 100 | 100 | 100 | 100 |
| acquisition time (*t_2_*) / ms | 41 | 15.4 | 10.3 | 10.3 |
| Decoupling sequence | Waltz64 | Spinal64 | Spinal64 | Spinal64 |
| ^1^H decoupling power / kHz | 5 | 90 | 90 | 90 |
| interscan delay / s | 2 | 3 | 3 | 3 |
| number of scans | 64 | 8 | 16 | 16 |

**Table S1 continued**

| **Sample** | C16-F | C16-P | C16-F | C16-P |
| --- | --- | --- | --- | --- |
| **Experiment** | ^13^C CP Hahn-Echo | ^13^C CP Hahn-Echo | ^13^C *T_1ρ_* | ^13^C *T_1ρ_* |
| **Figure** | 7, S11 | 7, S11 | 7, S11 | 7, S11 |
| ν_r_ / kHz | 17.0 | 17.0 | 17.0 | 17.0 |
| *B*_0_/ T | 11.7 | 11.7 | 16.4 | 16.4 |
| VTU / K | 250 | 250 | 272 | 272 |
| Probe | 3.2mm triple resonance | 3.2mm triple resonance | 3.2mm triple resonance | 3.2mm triple resonance |
| transfer I | ^1^H-^13^C CP | ^1^H-^13^C CP | ^1^H-^13^C CP | ^1^H-^13^C CP |
| *ν*_1_(^1^H) / kHz | 60.0 | 60.0 | 60.0 | 60.0 |
| *ν*_1_(^13^C) / kHz | 42.6 | 42.6 | 45.9 | 45.9 |
| Shape | Tangent ^1^H | Tangent ^1^H | Tangent ^1^H | Tangent ^1^H |
| time / ms | 0.7 | 0.7 | 0.7 | 0.7 |
| Spinlock / kHz | - | - | 45 | 45 |
| *t_2_* increments | 1024 | 1024 | 1024 | 1024 |
| spectral width (*t_2_*) / kHz | 100 | 100 | 100 | 100 |
| acquisition time (*t_2_*) / ms | 5.1 | 5.1 | 5.1 | 5.1 |
| Decoupling sequence | Spinal64 | Spinal64 | Spinal64 | Spinal64 |
| ^1^H decoupling power / kHz | 90 | 90 | 90 | 90 |
| interscan delay / s | 2.7 | 2.7 | 2.7 | 2.7 |
| number of scans | 8 | 8 | 8 | 8 |

**Table S1 continued**

| **Sample** | C16-F | C16-P | C2-F |
| --- | --- | --- | --- |
| **Experiment** | ^1^H *T_1ρ_* | ^1^H *T_1ρ_* | ^1^H-^13^C CP-MAS |
| **Figure** | 7, S11 | 7, S11 | S1 |
| ν_r_ / kHz | 17.0 | 17.0 | 17.0 |
| *B*_0_/ T | 16.4 | 16.4 | 16.4 |
| VTU / K | 272 | 272 | 272 |
| Probe | 3.2mm triple resonance | 3.2mm triple resonance | 3.2mm triple resonance |
| Spinlock / kHz | 45 | 45 | - |
| transfer I | ^1^H-^13^C CP | ^1^H-^13^C CP | ^1^H-^13^C CP |
| *ν*_1_(^1^H) / kHz | 60.0 | 60.0 | 60.0 |
| *ν*_1_(^13^C) / kHz | 45.9 | 45.9 | 40.8 |
| Shape | Tangent ^1^H | Tangent ^1^H | Tangent ^1^H |
| time / ms | 0.7 | 0.7 | 0.9 |
| *t_2_* increments | 1024 | 1024 | 3072 |
| spectral width (*t_2_*) / kHz | 100 | 100 | 100 |
| acquisition time (*t_2_*) / ms | 5.1 | 5.1 | 15.4 |
| Decoupling sequence | Spinal64 | Spinal64 | Spinal64 |
| ^1^H decoupling power / kHz | 90 | 90 | 90 |
| interscan delay / s | 2.7 | 2.7 | 2.7 |
| number of scans | 8 | 8 | 40 |

**Supplementary reference**

1. Hediger S, Meier BH, Narayanan DK, Bodenhausen G, Ernst RR (1994) NMR cross polarization by adiabatic passage through the Hartmann-Hahn condition (APHH). Chem. Phys. Lett. 223:283–288.
